# Identifying hybrids & the genomics of hybridization: Mallards & American black ducks of Eastern North America

**DOI:** 10.1002/ece3.4981

**Published:** 2019-02-27

**Authors:** Philip Lavretsky, Thijs Janzen, Kevin G. McCracken

**Affiliations:** ^1^ Department of Biological Sciences University of Texas at El Paso El Paso Texas; ^2^ Department of Biology University of Miami Coral Gables Florida; ^3^ Department of Ecological Genomics, Institute for Biology and Environmental Sciences Carl von Ossietzky Universität Oldenburg Oldenburg Germany; ^4^ Department of Marine Biology and Ecology, Rosenstiel School of Marine and Atmospheric Sciences University of Miami Miami Florida; ^5^ Human Genetics and Genomics Hussman Institute for Human Genomics, University of Miami Miller School of Medicine Miami Florida; ^6^ Institute of Arctic Biology and University of Alaska Museum University of Alaska Fairbanks Fairbanks Alaska

**Keywords:** ddRADseq, evolution, haplotype blocks, hybridization, introgression, junctions, population genetics, speciation

## Abstract

Resolving evolutionary relationships and establishing population structure depends on molecular diagnosability that is often limited for closely related taxa. Here, we use 3,200 ddRAD‐seq loci across 290 mallards, American black ducks, and putative hybrids to establish population structure and estimate hybridization rates. We test between traditional assignment probability and accumulated recombination events based analyses to assign hybrids to generational classes. For hybrid identification, we report the distribution of recombination events complements ADMIXTURE simulation by extending resolution past F4 hybrid status; however, caution against hybrid assignment based on accumulated recombination events due to an inability to resolve F1 hybrids. Nevertheless, both analyses suggest that there are relatively few backcrossed stages before a lineage's hybrid ancestry is lost and the offspring are effectively parental again. We conclude that despite high rates of observed interspecific hybridization between mallards and black ducks in the middle part of the 20th century, our results do not support the predicted hybrid swarm. Conversely, we report that mallard samples genetically assigned to western and non‐western clusters. We indicate that these non‐western mallards likely originated from game‐farm stock, suggesting landscape level gene flow between domestic and wild conspecifics.

## 1 INTRODUCTION

Establishing population structure, resolving evolutionary relationships, and prioritizing conservation efforts depend on molecular diagnosability of individuals to their respective taxon. This is often complicated when dealing with recent radiations in which substantial genomic variation is shared due to ancestry and/or gene flow (Nosil, Harmon, & Seehausen, 2009; Nosil & Schluter, 2011; Orr, Masly, & Presgraves, 2004; Seehausen, 2004; Via, 2009; Wu & Ting, 2004). In particular, gene flow between taxa without sufficient pre‐ or post‐zygotic isolation can have substantial amalgamating effects, including species loss (Eckert & Carstens, 2008; Lenormand, 2002; Nosil, Funk, & Ortiz‐Barrientos, 2009; Petit & Excoffier, 2009; Samuk et al., 2017). Determining the frequency of gene flow, and its geographic reach, is an essential step toward understanding the effect of hybridization on species and the speciation process, in general. Whereas gene flow may be predicted to occur because of the identification of hybrids, this does not necessarily establish the occurrence of gene flow, which requires subsequent backcrossing to effectively move genetic material between taxa (Slatkin, 1985; Vila, Seddon, & Ellegren, 2005). Thus, hybridization itself may not pose a genetic threat if hybrids do not or rarely backcross back into their parental population(s) (Todesco et al., 2016).

Among birds, the order Anseriformes, which includes ducks, geese, and swans, exhibits some of the highest rates of hybridization (Grant & Grant, 1992; Scherer & Hilsberg, 1982), with hybrids denoted among almost all pairwise comparisons within geese or ducks (Johnsgard, 1960; Ottenburghs et al., 2017; Ottenburghs, Ydenberg, Hooft, Wieren, & Prins, 2015). Among them, the mallard complex—comprised of 14 taxonomic units of mallard‐like ducks found around the world (Lavretsky, McCracken, & Peters, 2014)—has been particularly complicated by hybridization (Lavretsky, Engilis, Eadie, & Peters, 2015; Lavretsky, Hernández Baños, & Peters, 2014). Importantly, the dichromatic mallard (*Anas* *platyrhynchos*) has come into secondary contact and readily hybridizes with many of the other mallard‐like species. In addition to the expansion of wild mallard populations, many feral or domesticated mallards are also annually released or escape, further increasing the chance of hybridization (Champagnon et al., 2013; Guay & Tracey, 2009; Lavretsky, Hernández Baños et al., 2014; US Fish & Wildlife Service, 2013). Here, we assess whether a century of secondary contact and hybridization between North American mallards and American black ducks (*A*. *rubripes*; “black duck”) has resulted in the hypothesized genetic extinction of the iconic eastern black duck (Mank, Carlson, & Brittingham, 2004), and to what extent interspecific gene flow has affected the genetic integrity of North America's eastern mallard population.

### 1.1 Study system

The history of secondary contact between North American mallards and black ducks has caused concern over the possible genetic extinction of black ducks (Rhymer, 2006; Rhymer & Simberloff, 1996). Specifically, while mallards are currently widespread across North America, they were rarely observed east of the Mississippi River prior to the 1950s (Johnsgard, 1967; Merendino & Ankney, 1994; Snell, 1986). Causes for the dramatic change in the geographic distributions of mallards have been attributed to direct augmentation by game managers, sportsmen, and others releasing ~500,000 captive mallards per year along the east coast since the 1920s, with large‐scale releases ending in the 1950s and 1960s (Hepp, Novak, Scribner, & Stangel, 1988; Heusmann, 1974; Soutiere, 1986); although a couple hundred‐thousand game‐farm mallards continue to be released today (USFWS, 2013). Additionally, conversion of boreal forests into open habitat due to changing agricultural practices led to the expansion of western mallard populations and dramatic increases in mallard abundance (~600%) east of the Mississippi River beginning in the 1950s (e.g., southern Ontario; Hanson, Rogers, & Rogers, 1949). Given this history, we predict that the North American mallard is likely the product of both recent natural invaders and domestic ducks (Osborne, Swift, & Baldassarre, 2010; USFWS, 2013), resulting in the presence of multiple genetic mallard groups in North American samples.

Concern over high rates of bi‐directional gene flow between mallards and black ducks, as well as with the other New World monochromatic taxa (Mexican (*A*. *p*. *diazi*) & mottled (*A*. *fulvigula*) ducks) primarily stemmed from early mitochondrial DNA (mtDNA) research. Specifically, the New World mallard clade is characterized by two divergent mtDNA haplo‐groups: Old world (OW) A and New World (NW) B (Ankney, Dennis, Wishard, & Seeb, 1986; Avise, Ankney, & Nelson, 1990; Lavretsky, Hernández Baños et al., 2014). Whereas Eurasian mallards largely possess OW A haplotypes, NW mallard clade taxa have significant representation of both OW A and NW B haplotypes (Avise et al., 1990; Johnson & Sorenson, 1999; Kulikova et al., 2005; Kulikova, Zhuravlev, & McCracken, 2004; Lavretsky, McCracken et al., 2014). Competing hypotheses regarding the cause for the presence of both major haplogroups, as well as observed paraphyly within New World taxa include the following: (a) historical secondary contact between New World (NW) monochromatic species with Eurasian mallards resulted in bi‐directional gene flow, (b) an ancestral mallard invaded and speciated throughout the NW and the present paraphyly is the result of incomplete lineage sorting within NW taxa, and (c) NW mallards and allies were monophyletic for the B haplotype, but more recent gene flow with occasional Eurasian mallards and/or influx of feral mallards (hypothesized to be of OW origin) resulted in mtDNA paraphyly. However, conclusively testing between these competing hypotheses has been stifled due to the inability to genetically identify individuals to species, and thus estimate true rates of hybridization and gene flow using bi‐parentally inherited nuclear markers.

Our primary objective is to determine the rate of hybridization and extent of gene flow between mallards and black ducks using high‐throughput DNA sequencing methods. Whereas hybrids have been well documented between mallards and black ducks in the wild, we aim to determine whether hybridization has resulted in gene flow, including whether backcrossing is unidirectional (toward either black ducks or mallards) or bi‐directional (toward both black ducks and mallards). We use two methods to identify hybrids (F1) and generational backcrosses (≥F2): (a) traditional approaches in estimating assignment probabilities across samples and (b) novel techniques that utilize information regarding local ancestry across chromosomal haplotype blocks to assign hybrid status (Janzen, Nolte, & Traulsen, 2018; Leitwein, Gagnaire, Desmarais, Berrebi, & Guinand, 2018). Comparing assignments between the two methods will determine whether traditional methods struggle to assign late generational hybrids that often possess only small fraction of the genome as admixed (Lawson, Dorp, & Falush, 2018). Additionally, we provide an empirical test to determine the utility of accumulated recombination analyses for species that are at the earliest stages of species divergence and largely differentiated by small frequency differences. If hybridization has resulted in extensive gene flow between species, then we expect to find few, if any “pure” individuals, warranting one or both species to be considered a hybrid swarm. Alternatively, if sufficient isolating mechanisms have built up between the two species, then we expect the majority of samples to be assigned with high probability to their respective species or first‐generation hybrids (F1), and little evidence of generational backcrosses (≥F2). Such a scenario would be consistent with the reinforcement hypothesis in which taxa retain species boundaries during secondary contact due to viability limitations of any potential hybrids (Servedio & Noor, 2003).

Next, if released game‐farm mallards established a viable feral population in Eastern North America, we expect to identify a unique genetic signature of such population structure in eastern mallards. Additionally, if feral mallards tended to breed with black ducks, then we also expect to find eastern black ducks—their closest abundant relative in the first part of the 20th century—with some assignment to a secondary, non‐western mallard population. Alternatively, if the presumed survival of released feral mallards is low (Osborne et al., 2010; USFWS, 2013), then we expect to find little or no indication of a second mallard population and thus, no evidence of gene flow from domestic mallard variants into wild populations of mallards or black ducks.

Finally, by genetically vetting sampled individuals as pure individuals, hybrids, or backcrossed hybrids, we aim to determine the effectiveness of the current set of phenotypic characters (Kirby, Reed, Dupuis, Obrecht, & Quist, 2000) used to assign individuals to species or establish a hybrid status. A recent study that genetically vetted phenotypic traits between mallards, mottled ducks (*A*. *fulvigula*), and their hybrids reported that a key character used to identify hybrids was in fact found in 10% of genetically “pure” mottled ducks (Bielefeld et al., 2016), suggesting that at least some of the phenotypic characters may not at all be entirely diagnostic. Thus, assessing genetic assignments will provide important information that will either validate current practices or identify which species‐cohort requires re‐evaluation. In addition, identifying discrepancies between per sample phenotypic and genetic assignment will allow us to compare and test the extent to which results are biased by incorrectly identified samples. In general, without genetically vetting a phenotypic trait, individuals may be incorrectly assigned to species, including the misidentification of a hybrid. Such a bias has the potential to impact downstream analyses and estimates of various summary statistics, rates of gene flow, evolutionary histories, etc., and perhaps resulting in skewed conclusions.

## 2 METHODS AND MATERIALS

### 2.1 Sampling, DNA extraction, library preparation, and de‐multiplexing

**Figure 1:**
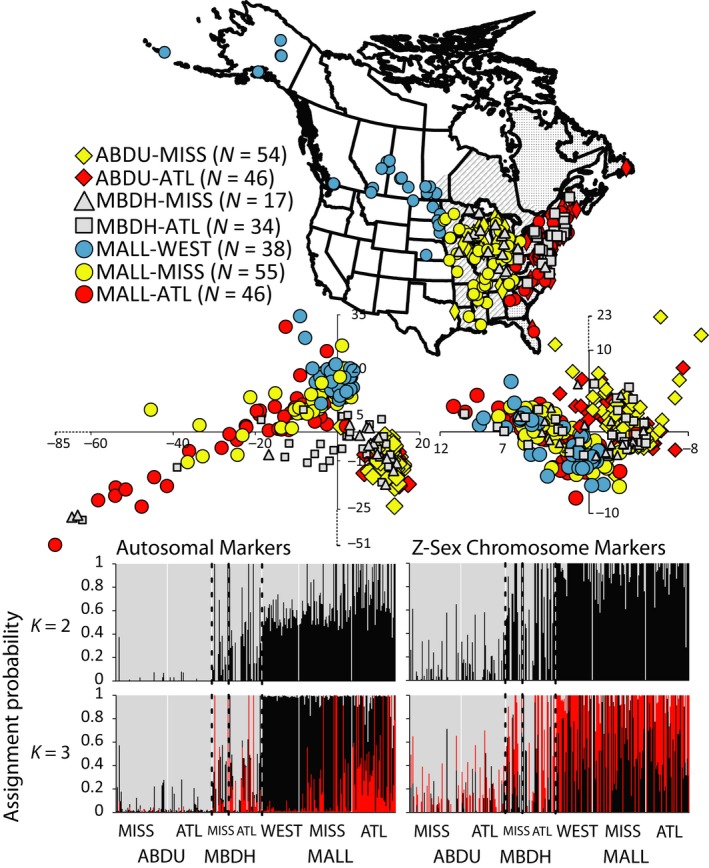
Map of sample locations for American black ducks (ABDU), mallards (MALL), and hybrids (MBDH)—taxonomic or hybrid assignments based on original USFWS phenotypic‐based assignments (also see Supporting Information Table S1; *N = *number of samples). The Mississippi flyway (MISS; striped) and Atlantic flyway (ATL; dotted) are denoted, with all areas west of the Mississippi River considered “WEST.” Scatter plots of PC1 (*x*‐axis) and PC2 (*y*‐axis) are plotted for 3,037 Autosomal (PC1 proportion of variance = 0.0092 [*SD* = 16.071] & PC2 proportion of variance = 0.00566 [*SD* = 12.61]) and 163 Z‐chromosome (PC1 proportion of variance = 0.023 [*SD* = 3.65] & PC2 proportion of variance = 0.019 [*SD* = 3.34]; also see Supporting Information Figure S3) ddRAD‐seq loci. Additionally, we present ADMIXTURE based maximum likelihood estimation of individual assignment probabilities for *K*population values of 2 and 3 based on autosomal or Z‐linked markers, respectively

Given our interest in determining the extent of hybridization between black ducks and mallards, we specifically targeted regions where the two cooccur (i.e., Mississippi and Atlantic flyways; Figure 1). A total of 290 samples were acquired across black duck, mallard, and their hybrid distributions in North America (Figure 1; Supporting Information Table S1), with the majority acquired at the 2010 U.S. Fish and Wildlife Services' (USFWS) flyway Waterfowl Wingbee meetings. For all samples, genomic DNA was extracted using a DNeasy Blood & Tissue kit following the manufacturer's protocols (Qiagen, Valencia, CA, USA). Extractions were quantified using a NanoDrop 2000 Spectrophotometer (Thermo Fisher Scientific Inc.) to ensure a minimum concentration of 0.02 µg/µl. Library preparation for multiplexing followed steps outlined in Lavretsky, Dacosta et al. (2015) (also see DaCosta & Sorenson, 2014). The samples were pooled in equimolar concentrations, and 150 base pair, single‐end sequencing was completed on an Illumina HiSeq 2500 at the Tufts University Core Genomics Facility. Raw Illumina reads have been deposited in NCBI's Sequence Read Archive (SRA; http://www.ncbi.nlm.nih.gov/sra; SRA data PRJNA516035).

Raw Illumina reads were demultiplexed and processed using the computational pipeline described by DaCosta and Sorenson (2014; Python scripts available at http://github.com/BU-RAD-seq/ddRAD-seq-Pipeline) and following steps outlined in Lavretsky, Dacosta et al. (2015). The pipeline clusters demultiplexed and filtered reads into putative loci based on sequence similarity and genomic position as determined by BLAST, aligns reads within each putative locus, and infers genotypes for individual samples at each locus. Final output files (e.g., fasta, ADMIXTURE) were generated with custom python scripts that set a higher minimum sequencing depth to score an allele (Lavretsky et al., 2016). To limit any biases due to sequencing error and/or allelic dropout, alleles with less than 5x coverage were scored as missing, such that a minimum of 10 reads were required to score a locus as heterozygous. Finally, loci with <20% missing genotypes were retained for downstream analyses. Chromosomal positions across markers were attained by aligning a reference sequence across ddRAD markers to the mallard genome (Kraus et al., 2011; Huang et al., 2013; chromosomal assembly provided by T. Farault, unpubl. data). Doing so allowed us to separately analyze autosomal and Z‐linked markers in all downstream analyses.

### 2.2 Mitochondrial DNA

Primers L78 and H774 were used to PCR amplify and sequence 653 bp of the mtDNA control region (Sorenson, Ast, Dimcheff, Yuri, & Mindell, 1999; Sorenson & Fleischer, 1996) following dideoxy sequencing methods described in Lavretsky, McCracken et al. (2014). The PCR products were sequenced on an ABI 3730 at the Yale University DNA Analysis Facility. Sequences were aligned and edited using Sequencher v. 4.8 (Gene Codes, Inc). All sequences have been submitted to GenBank (*accession* *numbers* MK425222–MK425495). The New World mallard clade is characterized by two divergent mtDNA haplo‐groups: Old world (OW) A and New World (NW) B (Ankney et al., 1986; Avise et al., 1990; Lavretsky, Hernández Baños et al., 2014). We evaluated and assigned mtDNA sequences of each sample to either the OW (A) or NW (B) haplogroup, and tested for longitudinal trends in haplogroup presence, as well as association with ddRAD nuclear‐based genetic assignments.

### 2.3 Population structure

Prior to estimating various descriptive statistics, we explored and visualized population structure using bi‐allelic SNPs with singletons (i.e., rare SNPs observed in only one individual) excluded and without a priori assignment of individuals to populations or species. Maximum likelihood estimates of population assignments for each individual were obtained with ADMIXTURE v.1.3 (Alexander & Lange, 2011; Alexander, Novembre, & Lange, 2009). Autosomal and Z‐linked SNPs were formatted for analyses using plink v. 0.67 (Purcell et al., 2007), following steps outlined in Alexander, Novembre, and Lange (2012). Updates to ADMIXTURE now permit for the effective analysis of sex‐linked markers (Shringarpure, Bustamante, Lange, & Alexander, 2016) without the increased concern of how heterogamy at sex chromosomes may impact results if homozygosity for all heterogametic samples is assumed by using the “–haplotype” function and designating the heterogametic sex. In birds, the female is the heterogametic sex (ZW), and the male is the homogametic sex (ZZ). Analyzing autosomal and Z‐linked markers separately, each ADMIXTURE v.1.3 analysis was run with a 10‐fold cross‐validation, and with a quasi‐Newton algorithm employed to accelerate convergence (Zhou, Alexander, & Lange, 2011). To limit any possible stochastic effects from single analyses, we ran 100 iterations at each population of *K* (from *K* of 1–10). Each analysis used a block relaxation algorithm for point estimation and terminated once the change (i.e., delta) in the log‐likelihood of the point estimations increased by <0.0001. The optimum K was based on the average of CV errors across the 100 analyses per K; however, additional Ks were analyzed for further population structure resolution. We then used the program CLUMPP v.1.1 (Jakobsson & Rosenberg, 2007) to determine the robustness of the assignments of individuals to populations at each K. First, the R program PopHelper (Francis, 2016) was used to efficiently convert ADMIXTURE outputs into CLUMPP input files at each K. In CLUMPP, we employed the Large Greedy algorithm and 1,000 random permutations. Final admixture proportions for each K and per sample assignment probabilities (Q estimates; the log‐likelihood of group assignment) were based on CLUMPP analyses of all 100 replicates per K. Additionally, population structure was also visualized using a Principal Component Analysis (PCA) in R (i.e., “prcomp”), with scoring of bi‐allelic SNPs as described by Novembre and Stephens (2008), which accommodate heterogamy when analyzing Z‐linked markers (also see Lavretsky, Dacosta et al., 2015).

Next, composite pairwise estimates of relative divergence (Φ_ST_), nucleotide diversity (π), and Watterson's θ for mtDNA, autosomal, and Z‐linked ddRAD‐seq loci were calculated in the R package PopGenome (Pfeifer, Wittelsbürger, Ramos‐Onsins, & Lercher, 2014) using concatenated datasets for each marker‐type and with indel positions treated as missing. If a substantial amount of samples showed discrepancies between the original phenotypic and genetic assignment (Supporting Information Table S1), then data were reanalyzed assuming original identifications by USFWS personnel and based on genetic assignments from ADMIXTURE analyses. These comparisons permitted us to test whether incorrect taxonomy (black duck <> mallard) or hybrid status (pure <> hybrid) based on phenotypic characters biased results.

### 2.4 Establishing hybrid indices & identifying hybrids

First, we employed methods outlined in Lavretsky et al. (2016) to simulate expected assignment probabilities for first‐generation hybrids (F1) and nine generations of backcrosses (F2–F10) into either the mallard or black duck parental population for ddRAD‐seq markers. In short, a total of ten F1 hybrids were first generated by randomly sampling an allele from the mallard and black duck gene pool across bi‐allelic SNP positions—each position was randomly sampled based on a probability proportional to the allelic frequency in each respective gene pool. Five hybrids were then backcrossed to either the mallard or black duck for nine generations. To limit potential biases in simulations, hybrid indices were reconstructed using only individuals with ADMIXTURE based probabilities of ≥95% assignment to either black duck or mallard. We ran a total of ten independent simulations, with data subsequently inputted into ADMIXTURE to estimate assignment probabilities for a *K* of 2 and 3. At each *K*, 25 iterations were run per simulation for a total of 250 ADMIXTURE outputs generated per *K,* which were then combined and converted in PopHelper (Francis, 2016) into CLUMPP input files. We employed the Large Greedy algorithm and 1,000 random permutations with final admixture proportions for each *K* and per sample assignment probabilities based on CLUMPP analyses of all 250 replicates per *K*. Per generation expected assignment probabilities were based on the average of either all ten (F1) or each of the five (F2–F10) backcrosses, along with each lower and upper limit.

### 2.5 Accumulated recombination events

Next, due to some potential limitations of likelihood or Bayesian methods (Lawson et al., 2018), samples were also categorized into hybrid and parental classes based on the number of accumulated recombination events. First, we followed recent methods to simulate the expected number of recombination events (termed “junctions”; Fisher, 1949, 1954) based on the idea that new junctions are formed when a crossover takes place at a site that is heterogenic for ancestry (Janzen et al., 2018). Subsequently, we measured the number of junctions in our samples and used this information to categorize each sample as parental or generational backcross. All analyses were based on the largest Chromosomes (1–7) as these provided the greatest number of markers (Supporting Information Figure S1), and thus, the highest likelihood of detecting junctions.

First, expectations of junctions across hybrid classes were simulated under two differing assumptions: (a) assuming a randomly mating hybrid swarm as done in Janzen et al. (2018), or (b) backcrossing with one parental species only (similar to above ADMIXTURE simulations). Under the first hybrid swarm scenario, we assumed a randomly mating hybrid swarm, where initial frequencies followed Hardy–Weinberg proportions and the initial heterozygosity for a first‐generation offspring of two randomly mating ancestors would be 0.5 (following Janzen et al., 2018). Simulations under the second assumption contrast to Janzen et al. (2018) because the backcrossing scheme used here does not impact the expected heterozygosity: over time the finite population size does not contribute to increased fixation of loci (see detailed proof in Supporting Information Appendix S1). Furthermore, although a finite number of markers might potentially impact results, for the application here the number of markers used is several orders of magnitude larger than the expected number of generations since the onset of admixture, in which case any limiting effects on having a finite number of markers can be safely ignored (see equation A11 in Janzen et al., 2018).

For empirical analyses, ddRAD data were transformed into a properly formatted input file for ANCESTRY_HMM (Corbett‐Detig & Nielsen, 2017) using a custom python script. ANCESTRY_HMM is a program that uses a Hidden Markov Model (HMM) to jointly infer local ancestry per SNP and age of the hybrid, given genetic data of the parental populations and the hybrid. However, ANCESTRY_HMM assumes a well‐mixed hybrid population, rather than a backcrossing population, and hence we opted to only use the inferred ancestry. Pilot runs showed that for some samples, extremely high hybrid age was inferred, causing an overestimation of the number of switches in ancestry (which are correlated with the age of the hybrid), and reducing overall confidence in local ancestry. This effect was most likely due to the relatively flat likelihood surface of hybrid age versus local ancestry. In order to avoid this problem, we modified the code of ANCESTRY_HMM by adding an exponential prior with a mean of 10 generations to the inferred generation time.

Inferring local ancestry across putative hybrid samples using ANCESTRY_HMM analyses requires SNPs from a potential parental pool from which allele frequencies are derived. First, an ancestry panel was created based on samples with ≥99% assignment to either black duck (*N* *=* 82) or mallard (*N* *=* 65) in ADMIXTURE analyses (i.e., assumed to be pure parental) (also see Leitwein et al., 2018). Using this ancestry panel, we applied ANCESTRY_HMM on the individuals within the ancestry panel as a cross‐validation, with the expectation that individuals used for the black duck panel were genetically 100% black duck, and individuals used for the mallard panel were genetically 100% mallard. Surprisingly, we found that of these individuals, only very few were 100% genetically black duck or mallard (28 out of 82 black ducks, and 6 out of 65 mallards), with many individuals showing at least one chromosome containing recombination event(s) suggesting the presence of “introgressed” genetic material. We therefore opted to use three different ancestry panels: (a) using the assignment based on ADMIXTURE analyses, (b) using only the 100% pure individuals as detected through ANCESTRY_HMM analyses, and (c) using all individuals that had at most one recombined chromosome (this yielded 64 out of 82 black ducks and 16 out of 65 mallards). Preliminary analyses (results not shown, pers. comm. Corbett‐Dettig) have shown that strong differences in panel sizes between species can potentially bias results, to mitigate this effect, we subsampled alleles from the more frequent species in order to obtain equal allele counts.

Using these ancestry panels, we inferred ancestry for each of the 143 potentially hybrid individuals separately across their respective chromosomes 1–7. Lacking a recombination map, we assumed a constant recombination rate across the chromosome, such that the relative distance between markers (e.g., the distance is base pairs divided by the total size of the chromosome) was equal to the relative recombination rate (e.g., if two SNPs are separated by 0.1% of all base pairs, then the recombination rate is 0.1 cM, assuming a chromosome size of 1 Morgan). Chromosome sizes were corrected for their total recombination rate, such that sizes for chromosomes 1–7 were 3.17, 2.26, 1.12, 0.93, 0.79, 1.20, and 0.98 Morgan, respectively (Huang *et* *al*., 2006). The total number of junctions per chromosome was determined by assessing the most likely ancestry within non‐overlapping windows along the chromosome; changes in most likely ancestry between windows were recorded as a junction. Pilot explorations showed that 20 windows per chromosome provided best results. Obtained results were visually verified to check against artefacts. Junction determination was performed blind, without prior knowledge about the inference of generation based on ADMIXTURE results. Given a number of observed junctions, we then obtained ten likelihood values across potential generations (F_1_–F_10_). The likelihood for each chromosome was calculated as the probability of observing *j* junctions after *t* generations, given the size of the chromosome in Morgan (e.g., Supporting Information Table S2). Then, given the number of observed junctions across the seven chromosomes, the full likelihood is the product of the seven separate likelihoods. In the absence of an analytical expectation for the likelihood of observing *j* junctions after *t* generations, instead, we used the observed frequency in simulations, based on 1,000 replicates. Standard errors of the mean frequency were very small, indicating that this approximation of the likelihood performs well.

To assess which generation fits the data best, we then calculated the AIC value, where we assumed one degree of freedom (*t*), such that: AIC = 2*df – 2 *log *P*(*t*) (Akaike, 1974), and where *P*(*t*) is the approximated likelihood as discussed above. Then, we calculated AIC weights (Wagenmakers & Farrell, 2004) to obtain the relative probability of the observed distribution of junctions being from that number of generations. The generation with the highest AIC weight was subsequently selected as the generation that best explained the data.

### 2.6 ANCESTRY_HMM simulations

To verify correctness of the junctions method and to test the power of the used markers we also performed simulations. We performed the same simulations as used to obtain approximate likelihoods, but instead of tracking junctions, individuals were artificially genotyped at each generation. In order to genotype individuals, we used the same SNP positions (relative along the chromosome) as used in the data, and determined “true” ancestry within the simulation for that individual at that position. Then, given true ancestry, the corresponding observed allele was drawn from the distribution of alleles as observed in the data. For example, for a SNP at location 0.1 cM, where in the data the observed allele counts are [40, 10] for black duck ([reference allele, alternative allele]) and [10, 40] for Mallard, then the corresponding allele (reference or alternative allele) is drawn from the matching distribution depending on the observed ancestry in the simulation. For example, if in the simulation the individual picked for “genotyping” is of ABDU ancestry at location 0.1 cM, an allele is drawn from the black duck distribution (i.e., [40, 10]).

After collecting alleles at the same SNP positions as in the data, the obtained alleles are analyzed using ANCESTRY_HMM to calculate local ancestry, and subsequently to count the number of junctions. Because the simulations only allow for the simulation of a single chromosome (rather than a full chromosome set of seven chromosomes), it is not possible to calculate expected hybrid status from the simulations, but instead we compared the inferred number of junctions compared to the expected number of junctions (based on the number of junctions observed in the simulations). Furthermore, we compared the degree of heterozygosity between simulated data, ancestry inferred from simulated data, and ancestry inferred from the empirical data. Doing so permitted us to determine whether there were any apparent biases toward certain ancestry. Finally, we repeated our analysis using a set of artificial SNPs where allele frequencies were artificially constructed to be strongly diagnostic (e.g., a scenario in which allele frequencies were differentially fixed between two species). We performed this analysis in order to quantify the expected uncertainty in hybrid status estimation, given high‐quality data.

### 2.7 Outlier analyses

We tested for statistical outliers that are putatively under selection with the program BayeScan v. 2.1 (Foll & Gaggiotti, 2008). BayeScan has a relatively low rates of false positives (<1%) for populations with low overall differentiation (Pérez‐Figueroa, García‐Pereira, Saura, Rolán‐Alvarez, & Caballero, 2010) as observed within the NW mallard clade (Φ_ST_ estimates range from 0.011 to 0.043; Lavretsky, Hernández Baños et al., 2014). Analyses included 20 pilot runs of 5,000 steps each, followed by 100,000 burn‐in and 100,000 sampling steps with a thinning interval of 10 for a total of 1,100,000 iterations. The prior odds parameter for the neutral model was set at 10, which equals log10 (PO > 1.0). We allowed a probability of false discovery (qval) of 0.05. Finally, to determine whether demographic and/or selective processes are the cause of outlier prominence, we calculated Tajima's D (Tajima, 1983), nucleotide diversity, and absolute divergence (i.e., *d_XY_*; Nei & Li, 1979) across markers in the R package PopGenome (Pfeifer et al., 2014). Specifically, for markers in which selection is the cause for high relative divergence (Φ_ST_), we expect a high estimate of absolute divergence, and a negative Tajima's D and low nucleotide diversity within the population that is most likely affected by directional selection.

## 3 RESULTS

We recovered 3,200 ddRAD‐seq loci that met our coverage and missing data criteria. Of those, 3,037 loci (362,644 base pairs; 68,187 single‐nucleotide polymorphisms [SNPs]) and 163 loci (19,873 base pairs; 2,511 SNPs) were assigned to autosomes and the Z‐chromosome, respectively (Supporting Information Figure S2). Marker coverage corresponded to chromosomal size (Supporting Information Figure S1). Final datasets comprised loci with an average median sequencing depth of 80 reads per locus per individual (median range = 24–241 reads/locus/individual), and on average, 98% (minimum of 86%) of alleles per individual per locus were scored. Finally, of the total ddRAD markers, 2,603 and 125 autosomal and Z‐linked markers, respectively, were successfully mapped to chromosomal position. Based on a genome size of 1.1 Gbp, marker density was every ~400 Kbp.

### 3.1 Population structure & hybrids

Both PCA and ADMIXTURE analyses for autosomal markers were based on 28,122 bi‐allelic SNPs, excluding singletons. The first two principle‐component axes in PCA analyses separated the majority of black duck and mallard samples, with more broad overlap across phenotypically identified hybrids (MBDH) (Figure 1; Supporting Information Figure S3). Several Mississippi and Atlantic flyway mallard samples, as well as putative hybrids made up a long tail within the autosomal PCA. Although ADMIXTURE analyses identified an optimum *K* of 1 (Supporting Information Figure S4), we explored other *K* values to test for additional resolution as such analyses tend to bias toward lower K values in cases of close ancestry (Janes et al., 2017; Lavretsky, Dacosta et al., 2015). First, *K* of 2 recovered black ducks as distinct from mallards. Next, *K* of 3 revealed a high degree of assignment to a second mallard group in the Mississippi and Atlantic flyways that corresponded to those samples in the long tail of the PCA analysis (Figure 1). We report no identifiable genetic structure within black ducks.

For Z‐chromosome markers, analyses were based on 638 bi‐allelic SNPs with singletons excluded. Both PCA and ADMIXTURE analyses (Optimum *K* of 2; Supporting Information Figures S2 and S3) differentiated between black ducks and mallards (Figure 1). There did not appear to be additional resolution at higher *K* values. The closer association in PCA analysis and lower resolution in ADMIXTURE results is likely the result of the 54‐fold difference in the number of analyzed SNPs. However, there was a significant correlation between Z‐chromosome and autosomal assignment probabilities (*R* ^2^ = 0.84, *p* < 0.0001) that provided confidence in attaining an overall genomic perspective using autosomal markers.

### 3.2 Hybrid simulations based on assignment probability

**Figure 2:**
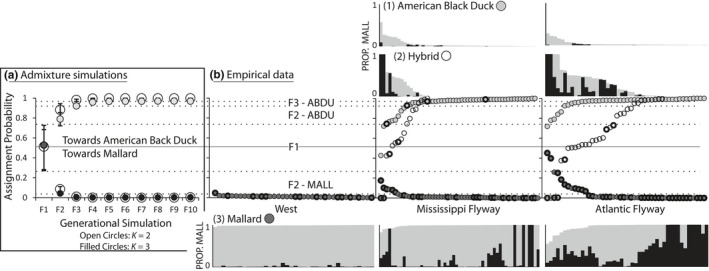
(a) The average and range of assignment probabilities from ADMIXTURE results at *K*of 2 and 3 across 25 simulated replications of hybridization (F1) and nine generations of backcrossing (F2‐F10) using genetically vetted American black ducks (ABDU) and mallards (MALL) (Supporting Information Table S1)—each *K*is based on 250 independent ADMIXTURE analyses. Simulations established assignment probability bins for parental American black ducks, mallards, F1 hybrids, two (F2‐ABDU & F3‐ABDU) categories for black duck backcrosses, and one (F2‐MALL) category of a mallard‐backcross (Supporting Information Tables S1 and S4). (b) Empirical data for western (WEST), Mississippi flyway (MISS), and Atlantic flyway (ATL) ABDU, MALL, and hybrid samples—taxonomic or hybrid assignments based on original USFWS phenotypic‐based assignments (also see Supporting Information Table S1). Above and below plotted assignments are corresponding ADMIXTURE assignment probabilities from *K*of 3 analyses across samples (Figure 1). Pure ABDU (no color) are denoted as having ≤5% assignment to the gray or black population. Pure MALL are denoted as having ≥98% assignment to and/or gray and black population(s). Note that western mallards are identified as a single (gray) population, and the prominence of the second (black) mallard population geographically increasing eastwardly. Finally, bold or non‐bold circles denote samples with Old World A or New World B mitochondrial haplogroups, respectively (Supporting Information Table S1)

Given the significant correlation between assignments from autosomal and Z‐linked markers, and the lower resolution for Z‐chromosome‐based ADMIXTURE results, we focused on autosomal markers to build expected hybrid indices to assign empirical samples to hybrid or pure classes. Moreover, with the novel structure recovered within eastern mallards, and interspecific assignments within phenotypically assigned black ducks being to western mallards (Figure 1), hybrid indices were simulated using black ducks and western mallards with ≥95% assignment at autosomal markers to their respective species. Within simulations, assignment probabilities between *K* runs were significantly correlated (*R* ^2^ > 0.99, *p* < 0.0001; *t* test *p* value = 0.78), however, they differed in final expected assignment probabilities. At *K* of 2, assignment probabilities eventually plateaued at ~99% assignment for backcrosses to their respective parental population. Whereas assignment probabilities for mallard‐backcrossed simulations still plateaued at ~99% assignment at *K* of 3, small interspecific assignments remained across generational classes when evaluating simulation for black duck backcrosses; although, F4‐F10 generations reached a consistent assignment probability of ≥95% to their black duck‐backcrossed parental population. Thus, while slight interspecific assignments may indicate hybrid status in empirical data, our simulations suggest that this may not be the case when evaluating *K* of 3 in our dataset (Lavretsky et al., 2016). Instead, the small interspecific assignment seen across black ducks (Figure 1) likely represent shared ancestry and perhaps forcing data into a population *K* of 3. Nevertheless, regardless of *K* value, lower and upper limits of expected assignment probabilities consistently overlapped one another for each respective species (Figure 2). In general, expected assignment probabilities during backcrossing differed, with a plateau in “purity” reached at the F3 versus the F4 stage for mallard or black duck backcrosses, respectively. Given the expected assignment probabilities for F1‐F3 generations and the “purity” cut‐offs set based on simulations for either black duck or mallard backcrosses (Figure 2; Table 1), we found that a proportion of samples phenotypically identified as black duck (MISS = 15%; ATL = 20%) and mallard (WEST = 3%; MISS = 16%; ATL = 26%) had hybrid ancestry. Similarly, only 65% and 62% of phenotypically identified hybrids in the Mississippi and Atlantic flyways, respectively, were genetically true hybrids. A large proportion of samples identified as hybrid were genetically assigned as “pure” mallards (MISS = 12%; ATL = 12%) or black ducks (MISS = 24%; ATL = 26%; Figure 2; Supplementary Table S1).

**Table 1:** Simulation‐based indices for “pure” black ducks, “pure” mallards, F1 hybrids, F2‐black duck and mallard backcrosses, as well as F3‐black duck backcrosses (Figure 2; Supporting Information Table S1). Per index, assignment probabilities are based on the proportion of intra‐ and inter‐specific assignment. Purity assignments based on percentage assigned to black duck populations. Regions include WEST (west of the Mississippi River), the Mississippi flyway, and Atlantic flyway (Figure 1), with the number of samples per region provided (*N*). The total number and proportion of the total per region recovered per group, as well as the percent of samples within each region and per group having ≥5% assignment to a secondary mallard population and mitochondrial (mtDNA) Old World A haplogroup are also provided (Figures 1 and 2)

Group	Index	WEST (*N = *38)	Mississippi flyway (*N = *126)	Atlantic flyway (*N = *126)
American Black Duck (ABDU)	PURE ≥95%	0	47 (0.37)	48 (0.38)
	Prop. Assigned to Secondary Mallard Group	NA	NA	NA
	Prop. A mtDNA haplogroup	NA	2 (0.043)	0
Hybrid (F1)	27% <F1 < 72%	0	9 (0.071)	18 (0.14)
	Prop. Assigned to Secondary Mallard Group	NA	5 (0.56)	14 (0.78)
	Prop. A mtDNA haplogroup	NA	2 (0.22)	4 (0.22)
F2 TOWARD ABDU	10% <F2 ≤ 27%	0	10 (0.079)	9 (0.071)
	Prop. Assigned to Secondary Mallard Group	NA	1 (0.10)	3 (0.33)
	Prop. A mtDNA haplogroup	NA	1 (0.10)	1 (0.11)
F3 TOWARD ABDU	5% <F3 ≤ 10%	0	3 (0.024)	3 (0.024)
	Prop. Assigned to Secondary Mallard Group	NA	0	0
	Prop. A mtDNA haplogroup	NA	1 (0.33)	0
Mallard (MALL)	PURE ≤2%	37 (0.97)	48 (0.38)	38 (0.30)
	Prop. Assigned to Secondary Mallard Group	3 (0.081)	19 (0.40)	35 (0.92)
	Prop. A mtDNA haplogroup	14 (0.38)	20 (0.42)	28 (0.74)
F2 TOWARD MALL	2% <F2 ≤ 27%	1 (0.026)	9 (0.071)	10 (0.079)
	Prop. Assigned to Secondary Mallard Group	0	5 (0.56)	10 (1.0)
	Prop. A mtDNA haplogroup	0	6 (0.67)	8 (0.80)

### 3.3 Hybrid simulations based on recombination junctions

**Figure 3:**
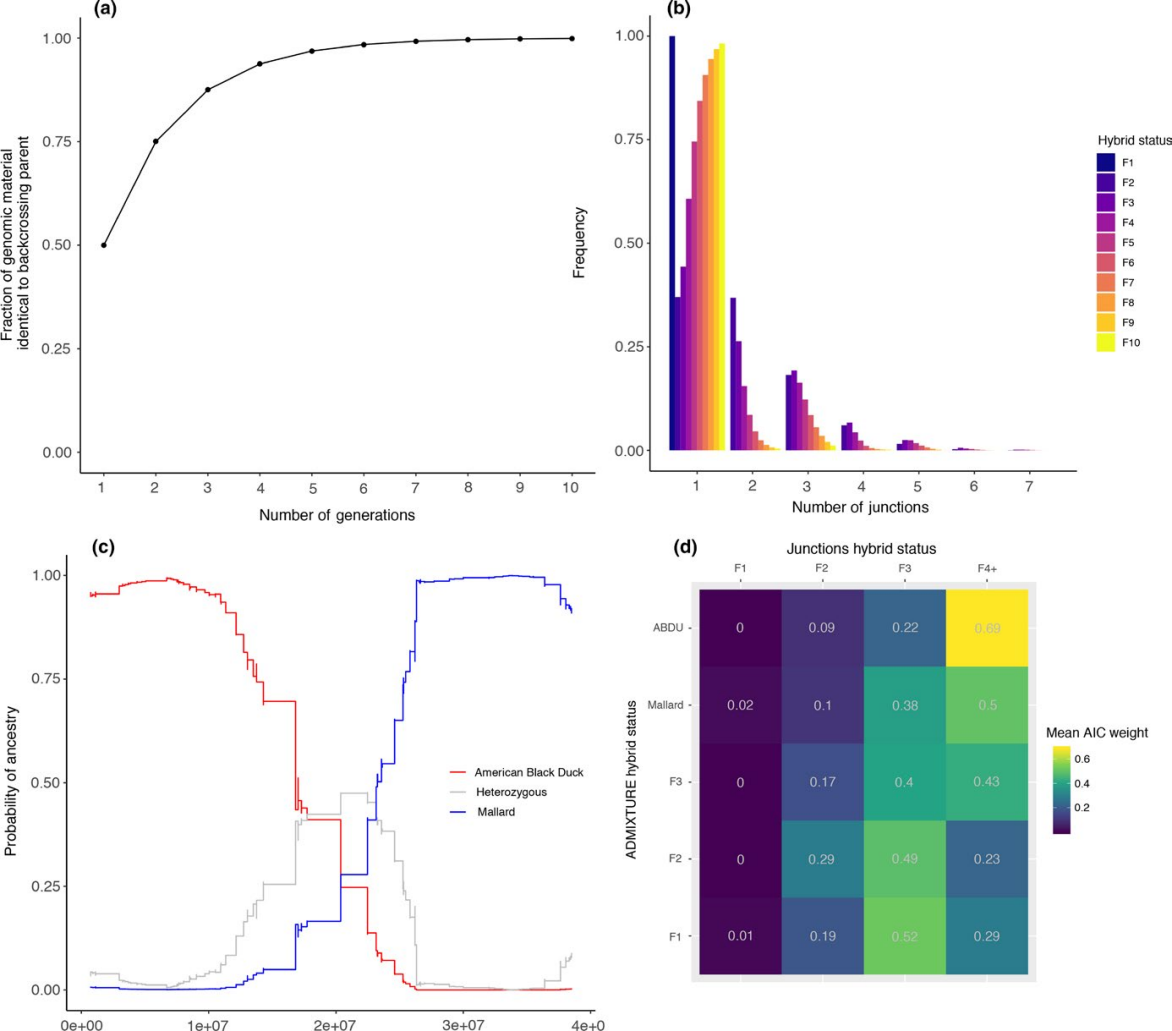
(a) Expected fraction of genomic material in the genome belonging to the backcrossing parent, as given by equation 2 (Supporting Information Appendix S1) (solid line), and matching simulation results (solid dots). (b) Observed frequency of junctions with increasing hybrid status. Values are averages over 1,000 replicates with *N* = 1,000, size of the chromosome is 1 Morgan. (c) Example of inferred ancestry probabilities along chromosome 7 for sample PL734, using the default ancestry panel. Ancestry was inferred using ANCESTRY_HMM, a red line indicates American black duck ancestry, a blue line indicates mallard ancestry and gray indicates heterozygosity. The chromosome shown contains one single junction (a small chromosome was chosen for demonstration purposes, to avoid a large number of junctions; see Supporting Information Figure S5 for all samples). (d) Average AIC weight support of the junctions approach (columns) for the different hybrid status classes assigned by the ADMIXTURE method (rows)

Although the genetic divergence between the two parental species is limited and few strongly diagnostic SNPs are apparent in the data, ANCESTRY_HMM was able to resolve local ancestry for all hybrid samples (Figure 3; Supporting Information Figure [Link], [Link]). Furthermore, most chromosomes had clear patterns of ancestry and recombination junctions, where ancestry changed from one type to another, interspersed with genomic areas having increased heterozygosity (Figure 3c; Supporting Information Figure S5). Although F1 individuals are expected to have chromosomes without any junctions and with excess heterozygosity, chromosomes without junctions always showed biased ancestry toward one of the parents (e.g., chromosomes 1–3 of sample PL010314; Supporting Information Figure S5). Thus, while using the number of accumulated recombination events (junctions) to independently assess hybrid status extended identification of hybrids into the F7 category, as compared to ADMIXTURE analyses, it did lack in detection of F1 hybrids.

We found a strong effect of the reference panel used. When only parents that contained pure ancestry were used, only few junctions were detected. However, given the lack of heterozygosity, these individuals were not inferred as F1 individuals (which would also lack junctions), but rather were assumed to be higher generation backcrosses (F4 and higher). The lack of detection of junctions is most likely due to the limited sample size of the reference panel, which makes detection of rare alleles problematic. Including parental individuals that showed at most one recombination event across seven chromosomes increased the ability to detect junctions, but still the match with ADMIXTURE analyses is low, and results tend to be biased toward higher generations. Found heterozygosity rates also reflected differences between the used reference panels; with the default reference panel being significantly different from both the pure and one‐recombination reference panels (Tukey's HSD pairwise comparison per chromosome, adjusted *p* < 0.0001), except for chromosome 5, where the default panel and the one‐recombination panel were not significantly different (*p* = 0.607, Tukey's HSD). The pure and one‐recombination panels yielded results not significantly different from each other (*p* > 0.1 for chromosomes 2–5), except for chromosomes 6 and 7, where they were significantly different (*p* < 0.0001).

**Figure 4:**
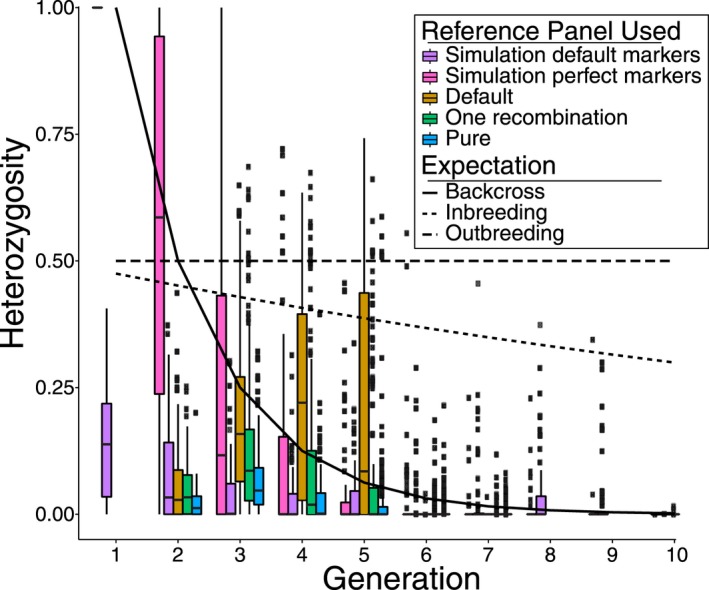
Observed heterozygosity across the different reference panels (“Default”, “One Recombination” or “Pure”). Furthermore, results of simulations using highly diagnostic markers (“Simulation perfect markers”) and simulations using the default panel are shown (“Simulation default markers”). The lines indicate the expected heterozygosity under a backcrossing scheme (solid line), outbreeding scheme (dashed line), or inbreeding scheme (*N* = 10) (dotted line). Please note that the boxplot of the first generation of “Simulation perfect markers” is represented as a single line

Observed heterozygosity across samples did not match any of the analytical predictions (Figure 4); under an outcrossing scheme (e.g., without gene flow from the parentals) we would expect the average heterozygosity to either remain constant if the hybrid population is large, or to decrease approximately linearly if the hybrid population is very small (*N* = 10 in Figure 4). In the data, we observe a slight upturn in heterozygosity around generations 3–4, but do not recover patterns as expected under either of the outcrossing scenarios. Similarly, under the backcrossing scheme we would expect heterozygosity to be high in the first few generations, to then to drop off exponentially. Although drop off is mimicked by the data from generation three onwards, we do not recover the excess heterozygosity in the first few generations. As a control, we find that we do recover heterozygosity rates similar to the backcrossing expectation in the simulations using perfect markers, but that this relationship breaks down when using markers from the empirical data. This shows that it is not the distribution of diagnostic markers that hinders detection of heterozygosity, nor the pipeline applied. Rather, it is the diagnostic power of the markers that results in an inability to detect heterozygosity sufficiently.

Comparing hybrid status assignment across reference panels uncovers another apparent bias: it seems that as the “purity” of reference individuals in the reference panel increases, inference of local ancestry is increasingly biased toward black duck ancestry, where using the “Pure” ancestry panel almost all individuals are recovered as higher generation backcrosses toward black duck. This reference panel effect is most likely due to the higher sample size in black duck (even though sample sizes were subsampled to recover similar allele counts for analysis), which makes detection of rare (and often diagnostic) alleles more likely.

Next, results obtained using a hybrid swarm mating scheme were similar to results obtained using the backcrossing scheme (Supporting Information Figure S6), although both schemes report very different hybrid status for chromosomes which show a lack of recombination. For individuals with many chromosomes lacking recombination (for instance chromosomes 1–3 of sample PL010314; Supporting Information Figure S5), we found striking differences between the two mating schemes. The backcrossing scheme consistently infers these individuals to be the result of recurrent backcrossing, where the lack of recombination along the genome is the result of continuously mating again with the same parent. Conversely, the hybrid swarm scheme infers these individuals to be young hybrids, of F2 hybrid status. Although it is impossible to obtain such a chromosome through meiosis of an F1 individual (unless there are no double‐strand breaks during meiosis, which is rare), it is even more unlikely that these are of higher hybrid status (F3 and higher). Thus, although the best fit of the hybrid swarm mating scheme is in some cases F2 hybrid status, the overall fit is poor.

Across all samples, we recovered identical hybrid status using the ADMIXTURE simulations and the junctions approach in 37% of all samples (using the default panel and the backcrossing scheme, Supporting Information Table S3A–B). However, using the junctions approach we never infer any individual to be F1, suggesting a potential bias toward over‐detection of junctions (i.e., F1 individuals are completely heterozygous, lacking any junctions). Ignoring F1 assigned individuals, agreement between the methods increases to 46% (Supporting Information Table S3). In general, comparing hybrid status assignment between ADMIXTURE simulations, we find that of the 39 individuals with F2 hybrid status as determined using ADMIXTURE, 38% (*N* = 15) were also assigned F2 by the junction simulations, and 36% (*N* = 14) were assigned to F3, suggesting again a potential bias. The remaining 25% (*N* = 10) were assigned to F4 and F5. Of the 6 individuals assigned F3 hybrid status using ADMIXTURE, 67% (*N* = 4) were also assigned F3 hybrid status using junction simulations, with the remaining individual being assigned F4 status. Of the 56 individuals assigned pure mallard ancestry, 27 individuals (47%) were assigned F3, and 24 individuals (41%) were assigned F4 or higher, indicating agreement between the two methods as ADMIXTURE is unable to distinguish ≥F3‐mallard backcrosses (Figure 2). The remaining 7 individuals (12%) were assigned F2 status. For individuals assigned black duck ancestry using ADMIXTURE simulations, agreement using the junctions simulations is much higher, with 10 out of 13 individuals (77%) assigned black duck ancestry as well, and 3 out of 13 individuals (23%) being assigned F3 hybrid status.

These results are all focusing on the maximum AIC weights, ignoring cases where AIC weights were perhaps relatively similar across hybrid statuses, indicating overall ambiguity in assignment. Comparing average AIC weights across assignments (Figure 3d), we find that the highest AIC weight most often matched ADMIXTURE assignments, and that generally, this AIC weight outweighed the others by a reasonable margin. For example, ABDU assigned individuals received an AIC weight of 0.69 in favor of being F4+, compared to an AIC weight of 0.22 of being F3 (and an even lower AIC weight for F2 & F1); a similar trend was for individuals assigned as “pure” mallard, AIC weight in favor of late generational backcrosses (F4+) exceeds that of the other hybrid statuses as well (0.5 vs. 0.38 and lower). Interestingly, AIC weight for F1 assignment, irrespective of ADMIXTURE simulations results, was extremely low, with AIC weights ranging from 0 to 0.02. This reflects the lack of fully heterozygous, non‐recombined chromosomes as detected by local ancestry. For individuals assigned F3 status by ADMIXTURE simulations, we find rivaling AIC weights, with on average 0.4 for F3, and 0.43 for F4+, indicating that these samples were relatively ambiguous, and although junctions assignment sometimes did not match ADMIXTURE assignment, AIC weight for F3 (matching) assignment was usually high. For individuals assigned F2 hybrid status by ADMIXTURE simulations, this discrepancy is much stronger, however, with an average AIC weight of 0.49 in favor of F3 assignment, with only an AIC weight of 0.29 for F2, suggesting a potential bias toward F3 assignment.

### 3.4 Mitochondrial DNA & the non‐western mallard

Only two genetically vetted black ducks from the Mississippi flyway possessed OW A mtDNA haplotypes. Conversely, all genetically assigned mallards had a significant proportion of samples with OW A mtDNA haplotypes, with the frequency of this haplogroup increasing eastward (Figure 2; Table 1). Similarly, F1 through F3‐black duck backcrosses showed an overall increasing presence of B haplotypes with each subsequent backcross, whereas F2‐mallard backcrosses had a very high proportion of samples with OW A haplotypes (Figure 2; Table 1).

Significantly associated (*R* ^2^ > 0.23, *p* value <0.0001), samples possessing an A haplogroup tended to have ≥5% assignment to a non‐western mallard group, particularly in the Atlantic flyway (Table 1). Focusing on mallards, western mallards were characterized by a substantial number of samples with A haplotypes but only 3% having ≥5% assignment to a non‐western mallard group (Figure 2). In contrast, ~40% of Mississippi flyway mallards possessed an A haplotype and/or significant assignment to a second mallard group, with the highest prevalence of samples with A haplotypes (74%) and/or assignment to a secondary mallard group (92%) found in Atlantic flyway mallards. Similar trends were found for F2‐mallard backcrosses in which either half or all samples had A haplotype and/or assignment to a secondary mallard group (Figure 2; Table 1).

### 3.5 Testing for biases & summary statistics

Given the discrepancy in the number of mallards, black ducks, and hybrids being identified based on phenotype or genetics, we calculated and compared indices with samples grouped by either their original phenotypic identities or genetic assignments. Despite ~20% of phenotypically identified black duck and mallard samples having some hybrid ancestry (≥ 5% mallard assignment), between species estimates for overall Φ_ST_ (*R* ^2^ > 0.99, *p* < 0.0001; *t* test *p* value = 0.92), nucleotide diversity (*R* ^2^ > 0.99; *t* test *p* value = 0.97), *d_XY_* (*R* ^2^ > 0.99, *p* < 0.0001; *t* test *p* value = 0.99), and per ddRAD‐seq locus Φ_ST_ (*R* ^2^ > 0.99, *p* < 0.0001; *t* test *p* value = 0.88) were all significantly correlated and statistically similar. Given these similar results across various test statistics, we focused on findings using genetically vetted datasets only.

**Figure 5:**
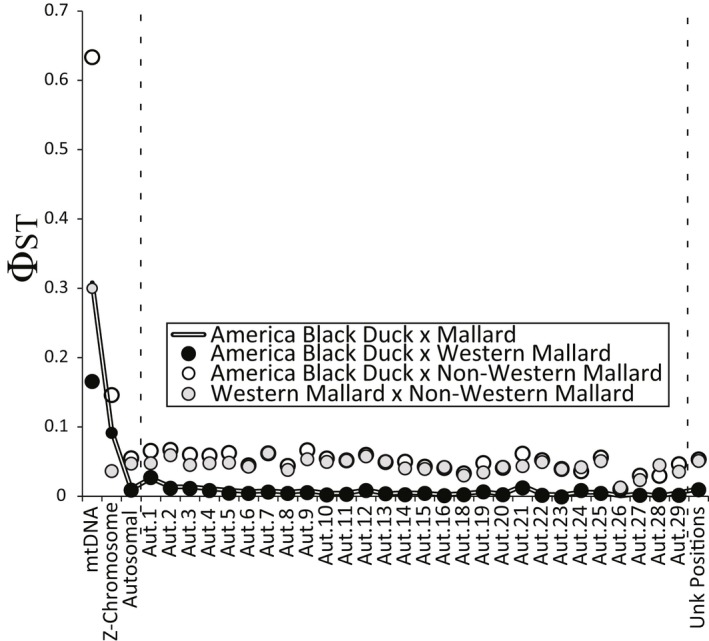
Per mitochondrial DNA (mtDNA) and chromosome composite pairwise Φ_ST_ estimates for genetically vetted mallards and American black ducks. Additional pairwise comparisons are done with genetically vetted western and non‐western mallards (Supporting Information Table S1)

In general, estimated differentiation (Φ_ST_) between genetically vetted black ducks and mallards was highest for mitochondrial (Φ_ST_ = 0.31) and Z‐chromosome (Φ_ST_ = 0.094) markers, with the lowest levels of differentiation for ddRAD‐seq autosomal markers (Φ_ST_ = 0.01; Figure 5). When dividing mallards into “pure” (≥98% assignment) western or non‐western groups, eastern mallards had overall elevated genomic differentiation compared to western mallards (composite Φ_ST_ across ddRAD‐seq = 0.047) and black ducks (composite Φ_ST_ across ddRAD‐seq = 0.057) (Figure 5). Composite Φ_ST_ estimates across ddRAD‐seq (0.010 vs. 0.057) and mtDNA (0.17 vs. 0.64) were four to six times higher as compared to those observed between western mallards and black ducks. Finally, western mallards and black ducks had similar estimates of nucleotide diversity and Watterson's θ across markers, which were on average 1.5‐times larger than those estimated for non‐western mallards (Supporting Information Table S4).

### 3.6 Genomic differentiation

**Figure 6:**
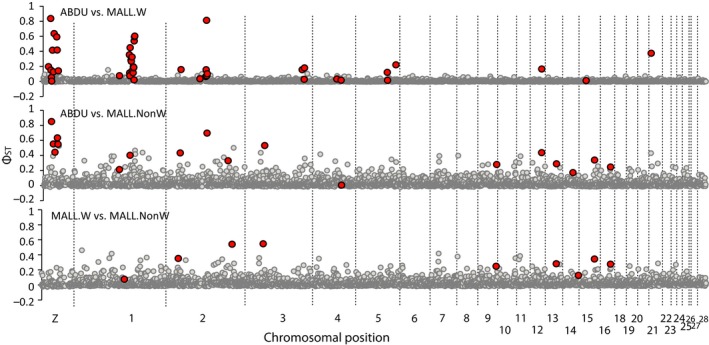
Chromosomally aligned Φ_ST_ estimates of 3,037 autosomal and 163 Z‐linked markers for pairwise comparisons between genetically vetted western mallards (MALL.W), non‐western (MALL.NonW) mallards, and black ducks (ABDU) (Supporting Information Table S1). Markers identified in BayeScan analyses as putatively under divergent selection are denoted in red

Φ_ST_across pairwise ddRAD markers were estimated and analyzed in BayeScan for signatures of divergent or balancing/purifying selection between genetically vetted western mallards, non‐western mallards, and black ducks (Figure 6). Between black ducks and western mallards, we found prominent Φ_ST_ peaks and signatures of divergent selection on the Z‐Sex (23 Mbp region between base pair positions 1.7E7 – 4.0E7)and three autosomal chromosomes (Chromosome 1 [31 Mbp region between base pair positions 8.9E7 – 1.2E8], Chromosome 2 (14 Mbp region between base pair positions 5.2E7 – 6.5E8), and Chromosome 21 [~2,155,252 base position]), as well as evidence of divergence selection on five other autosomal chromosomes (Chromosome 3 [1.6 Mbp region around ~1.0E8 base position], Chromosome 4 [5 Mbp region around ~4.6E8 base position], Chromosome 5 [13 Mbp region around ~5.0E8 base position], Chromosome 12 [~16E6 base position], and Chromosome 15 [~5.5E6 base position]). When comparing calculated Tajima's D, nucleotide diversity, and absolute divergence for putative outlier and non‐outlier markers (Supporting Information Figure S6), we first report that none of the outliers were explained by the highest absolute divergence; however, this is likely a poor proxy given the strong correlation with nucleotide diversity (Martin, Davey, & Jiggins, 2015). Nevertheless, we recover negative Tajima's D and low nucleotide diversity for particular outliers in either black ducks or western mallards. For example, prominent outlier regions on the Z‐Sex chromosome, Chromosome 1, and Chromosome 2 are best explained by low nucleotide diversity and negative Tajima's D in mallards as compared to black ducks (Supporting Information Figure S7), and infer this to suggest that these regions may harbor genes under divergent selection within the mallard lineage.

Once again, genetically vetted non‐western mallards showed genomes with elevated estimates of differentiation when compared to either black ducks or western mallards (Figures 4 and 5), and statistically different from comparisons between black ducks and western mallards (*R* ^2^ = 0.62; *t* test *p* value <0.0001). Furthermore, BayeScan analysis with non‐western mallards only identified several chromosomal regions that were consistent with divergent selection when compared against black ducks or western mallards (Figure 6), and which were completely absent in the black duck and western mallard comparison. In fact, of the 12 outlier markers recovered in comparison with non‐western mallards, 8 are shared when compared to either black ducks or Western mallards, including mapped locations: Chromosome 2 (~15,921,703 base position), Chromosome 3 (~22,353,205 base position), Chromosome 9 (~25,416,377 base position), Chromosome 13 (~16,132,845 base position), Chromosome 15 (~13,510,903 base position), and Chromosome 16 (~12,806,640 base position). This is contrast to 6 (of 6) Z‐Sex linked and 5 (of 16) autosomal outliers identified between black ducks and non‐western mallards that were also recovered when comparing black ducks and western mallards. Thus, those markers recovered when comparing black ducks or western mallards to non‐western mallards suggests that these outliers are the result of demographic or selective processes within non‐western mallards. Finally, we find an overall genomic shift toward positive values of Tajima's D in outlier and non‐outlier markers within non‐western mallards. This is in comparison to black ducks (Supporting Information Figure S8) and western mallards (Supporting Information Figure S9), which had a more even distribution of Tajima's D values across the genome, and largely negative values for outlier markers. Although, no significant outliers on the Z‐Sex Chromosome were found when comparing western and non‐western mallards, comparing black ducks and non‐western mallards demarcated markers within the same outlier region as within western mallards (Figure 6), which showed relatively lower nucleotide diversity in the non‐western mallard (Supporting Information Figure S8).

**Figure 7:**
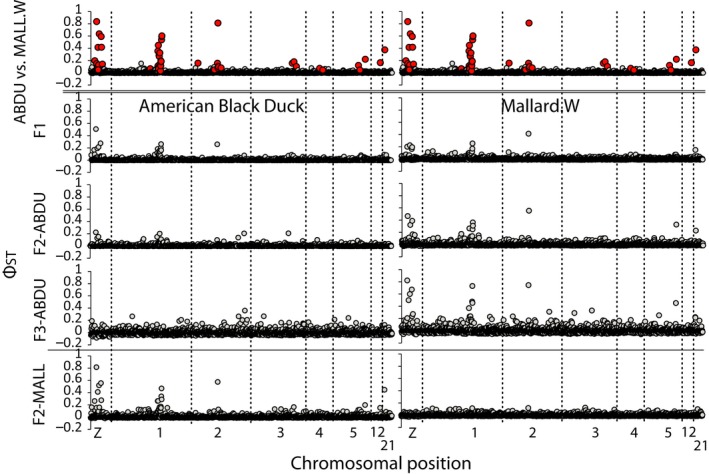
Aligned pairwise Φ_ST_ estimates of ddRAD markers for chromosomes that were identified to have non‐neutral regions between genetically vetted black ducks (ABDU) and western mallards (MALL.W)—Markers identified in BayeScan analyses as putatively under divergent selection are denoted in red in first Manhattan plot. Comparisons between genetically vetted black duck and western mallard samples with each hybrid class (Supporting Information Table S1) are presented

Focusing on chromosomes harboring statistical outliers, we compared pure black ducks and western mallards to each of the hybrid indices to determine if any particular chromosomal region showed signs of lower levels of introgression (Figure 7). In general, when compared against black ducks, outlier regions on the Z‐chromosome and Chromosome 1 showed a steady decay in differentiation across outlier markers with F3‐black duck backcrossed birds being genetically similar to pure black ducks (Figure 7). A reverse effect was seen when comparing mallards to each of the black duck‐backcrossed groups. In contrast, the F2‐mallard‐backcrossed group showed low levels of differentiation across ddRAD loci as pure western mallards and statistically similar estimates whether black ducks are compared to these backcrosses or pure mallards (*R* ^2^ = 0.87 *p* < 0.001; *t* test *p* value = 0.16). In contrast, outlier markers/regions on Chromosome 2 (~65,815,089 base position), Chromosome 12 (position ~16,329,258 base position), and Chromosome 21 (position ~2,155,252 base position) showed near identical differentiation across all black duck backcrosses when compared to mallards as between black ducks and mallards. These loci were undifferentiated when comparing mallards and F2‐mallard backcrosses or black ducks to ≥F2 black duck backcrosses.

## 4 DISCUSSION

### 4.1 Assigning hybrids: assignment probabilities versus junctions

Here, we used two separate simulation methods to infer hybrid status for all black duck, mallard, and putative hybrid samples. First, we simulated allele sorting during backcrossing, and maximum likelihood assignment probabilities with the program ADMIXTURE. Secondly, we inferred local ancestry along chromosomes 1–7 and used the distribution of recombination events across these chromosomes to infer hybrid status, following the theory of junctions (Janzen et al., 2018). Across these two methods, we find congruence for at least 37% of all samples and report limitations in both analyses. First, ADMIXTURE simulations were only able to resolve up to F3/F4 generation as compared to junction simulations that resolved hybrid status up to F9. These results confirm that traditional population assignment programs are reliable in determining several generations of hybrids but can miss‐assign late generational hybrids as parental (Leitwein et al., 2018). Conversely, junction simulations were unable to detect early hybrids (i.e., F1 hybrids) and were biased toward F3 hybrid status. Thus, while using the distribution of recombination events complements ADMIXTURE simulation by extending resolution past F4 hybrid status, we caution interpretations and hybrid assignment based on junctions for recently radiated taxa in which shared variation appears to limit hybrid identification. Most striking and concerning was the inability to identify F1 hybrids. In theory, the chromosomes of true F1 hybrids is comprised of one chromosome provided by each parental lineage, which results in excessive rates of heterozygosity across sites (Leitwein et al., 2018). It is possible that detecting recombination events (or in this case, the lack thereof) was problematic for F1 hybrids because the software used to infer local ancestry was not designed for a backcrossing scheme (ANCESTRY_HMM; Corbett‐Detig & Nielsen 2017). However, analyses on artificial data show that most likely the low degree of differentiation and resulting SNPs without sufficient diagnostic qualities between the two parental species causes the inability to detect excess heterozygosity by the software. Alternatively, the shortage of heterozygosity may indicate that perhaps hybridization may not be prevalent and thus, there really are few F1 hybrids and early backcrosses. Although possible, the latter scenario is unlikely given the clear assignment of F1 hybrids in ADMIXTURE analyses (Figures 1 and 2) and observed levels of hybridization between mallards and black ducks. Additionally, we find a strong effect of the reference panel used in ANCESTRY_HMM analyses (Supporting Information Tables S1 and S2). For example, restricting the reference panels to include only 100% genetically “pure” individuals caused a strong bias toward black duck ancestry when inferring local ancestry, leading to a loss of the ability to detect junctions. This seems to indicate that for systems in which few diagnostic SNPs exist, as the case for very closely related taxa, the detection of rare alleles requires a large reference panel to work accurately. Future work will require full genome sequencing of parental and putative generational hybrid individuals to fully understand whether the potential limitations based on junction numbers and heterozygosity from ddRAD markers is a result of methodology or a true biological pattern.

Hybrid status assignment assuming a hybrid swarm mating scheme (following Janzen et al., 2018), or using an exclusive backcrossing scheme were similar (Supporting Information Tables S1 and S2), barring some striking differences for individuals with highly purified chromosomes, where ancestry along the chromosomes was strongly biased toward one of the parents and crossovers were lacking. The backcrossing scheme resolved such genomic patterns by inferring that these individuals are the result of many consecutive generations of backcrossing. Alternatively, the hybrid swarm mating scheme could only resolve such genomic patterns by assuming that these were very young (F2) hybrids, having experienced very little recombination events (Supporting Information Table S1). However, although the hybrid swarm mating scheme inferred F2 to be the most likely hybrid status for these individuals (higher hybrid status would imply even more recombination events), F2 hybrid status in itself also seems unlikely, as this implies that both F1 parents experienced no recombination whatsoever during meiosis, often on multiple chromosomes. Therefore, these patterns seem to reinforce the idea that some of the hybrids analyzed are the result of repeated backcrossing with one of the parental species. Furthermore, the existence of these highly non‐recombined individuals seems to validate our intuition that the hybrids are backcrossing with one of the parental species. Although recombinatory simulations identified more backcrossed stages (up to F7) as compared to ADMIXTURE simulations (up to F3), both suggest that there are relatively few backcrossed stages before a lineage's hybrid ancestry is lost and the offspring are effectively genetically parental again. In general, we find an exponential decrease in hybrid assignment, with each subsequent backcross, thus becoming increasingly indistinguishable from its backcrossed parental population (Figures 2 and 3a). Thus, although our presumed scenario of backcrossing into a single population is un‐vetted with field observations, congruence across analyses regarding the prevalence of few backcrossed generations suggests that this scenario may represent the majority rule in which backcrossing occurs with the parental that is most geographically prevalent. In fact, our results support breeding experiments in which backcrossing into black ducks resulted in indistinguishability of offspring and the parental population ≥F3 stage (Kirby, Sargeant, & Shutler, 2004).

Summarizing, we find that for the black duck‐mallard system the recombinatory analyses were difficult to apply and might have provided misleading results and need to be applied with caution to other taxa with largely conserved genomes. Low differentiation between the two species caused a lack in diagnostic SNPs, which in turn made it difficult to detect junctions across the genome. Furthermore, low density of diagnostic SNPs lead to an underestimation of heterozygotic ancestry, which disqualified this method to detect F1 individuals. Although recombinatory analyses provide a promising future avenue to detect hybrid status of individuals as more molecular data is obtained, we conclude that the current dataset based on ddRAD sequences appears to provide too unreliable results, and we subsequently base our conclusions on ADMIXTURE results.

### 4.2 The genomics of mallards and black ducks in Eastern North America

We present the most comprehensive molecular study of mallards and black ducks to date, and are able to differentiate between these two previously closely related and genetically undiagnosable species (Figures 1 and 2; Lavretsky, Hernández Baños et al., 2014). First, whereas recent work identified migratory structure within black ducks (Lavretsky, Miller, Bahn, & Peters, 2014), we report no identifiable genetic structure within black ducks and suggest this species to be treated as a single genetic population. Conversely, we find evidence for two genetically distinct mallard stocks (Figures 1 and 2), which we characterize as western and non‐western. The North American mallard's history is known to be potentially complicated by the well documented and intense release of game‐farm mallards on the east coast since the early part of the 20th century (USFWS, 2013). Until recently, molecular methods did not distinguish among mallards across their Holarctic distribution (Kraus et al., 2013). However, Söderquist et al. (2017) used genomic sequencing and reported feral European mallards not only have a distinct genetic signature, but also showed them to have a significant impact on wild European mallard stocks. Although the authors did not find the same trend in North America, they did not adequately sample east of the Mississippi River and thus likely missed the non‐western genetic signature captured here. Unfortunately, without the presence of known game‐farm/feral mallards in our dataset, we are unable to formally test whether the non‐western mallard signal is the result of a game‐farm parental population. However, even without a known feral parental population to compare to, the genetic diagnosability of many of the geographically eastern samples strongly suggests these to be of domestic ancestry.

We provide strong evidence that supports differing evolutionary histories of samples genetically assigned as non‐western mallards (Supporting Information Table S1) when compared to western mallards or black ducks, including the following: (a) overall elevated genomic differentiation (Figure 5), (b) high frequency of OW A haplotypes (Figure 2), (c) a unique set of markers identified as putatively under selection (Figure 6), and (d) a genomic shift toward positive Tajima's D values as compared to black ducks (Supporting Information Figure S8) or western mallards (Supporting Information Figure S9). Finally, when analyzing “pure” non‐western mallards (≥ 98% assignment; Figures 1 and 2), we find these to have approximately half the calculated effective population size of western mallards or black ducks (black ducks (Avg. Watterson's θ_Aut_ = 0.013, Watterson's θ_Z_ = 0.0047); western mallards (Avg. Watterson's θ_Aut_ = 0.012, Watterson's θ_Z_ = 0.0047); non‐western mallards (Avg. Watterson's θ_Aut_ = 0.0082, Watterson's θ_Z_ = 0.0029; Supporting Information Table S4). In general, the lower genetic diversity coupled with the positive shift in Tajima's D values in non‐western mallards are likely signatures of bottlenecking that is often experienced during domestication (Innan & Kim, 2004; Makino et al., 2018; Tufto, 2017). Together, these results strongly support these non‐western mallards to be of alternative stock, and likely the result of a century of releasing game‐farm mallards in Eastern North America.

Given that all collected samples were taken from wild birds, and not from shooting preserves, our data strongly suggest that released game‐farm mallards have established a viable wild [feral] population that is significantly contributing to the genetics of their wild [native] counterparts. We also note that two female mallards (from Ohio) for which we obtained full bodies displayed male‐ or feral‐like phenotypic characters (e.g., green in head, white neck‐ring, red breast) and were identified as “pure” non‐western mallards (assignment probability of >99%), as well as carried A (OW) haplotypes. These samples suggest that feral female mallards are successfully surviving on the landscape. How gene flow from these non‐western, putatively feral birds affect fitness of wild populations remains to be determined; however, if unabated, the chance of negative impact(s) on wild populations may be significant and requires careful consideration (Randi, 2008; Tufto, 2017).

Although feral mallards pose a genetic threat to black ducks, we report overall lower levels of non‐western population assignment and OW A haplotypes in black ducks (Figure 2; Supporting Information Table S1). Conversely, 50%–100% of samples identified as first‐generation mallard‐backcrossed samples had substantial nuclear assignment to the non‐western mallard group and carried the OW A mtDNA haplotype (Table 1). Together, these data suggest that hybrids tend to backcross with mallards, and that these mallard backcrosses are largely into the non‐western or “feral” mallard population (Figure 2). The proximate cause for such a scenario remains to be determined. However, reduced representation of non‐western assignment and OW A haplotypes in black ducks (i.e., tendency of F1 black duck x feral hybrids to backcross into black ducks) may be due to black ducks showing assortative mating, in general, or simply tend to not overlap the primary habitat used by feral mallards (Osborne et al., 2010). Specifically, feral mallard success to adapt to human‐disturbed habitat (Diefenbach & Owen, 1989; Maisonneuve et al., 2006; Rogers & Patterson, 1984) and the black duck's natural reclusiveness and evasion of human‐disturbed habitats (Hepp et al., 1988) may simply limit the chance of contact between black ducks and feral mallards. Additionally, we hypothesize that mallard‐black duck hybrids may be using human‐dominated landscapes more often than their wild parentals, and thus are primarily coming into contact with other feral mallards (e.g., Hubb's Principle; Hubbs, 1955). Future conservation efforts will benefit from understanding the extent of gene flow from these putatively feral mallard birds into black ducks and native, wild mallards.

### 4.3 Old world mtDNA haplotypes are associated with the non‐western mallard group

**Figure 8:**
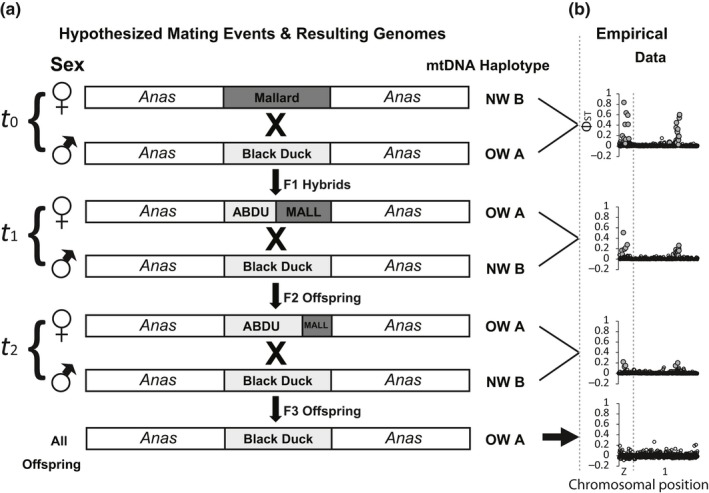
Hypothesized mating between mallards and black ducks (time *t*_0_), including subsequent backcrosses (times *t*_1–2_) into American black ducks with expected genomic contribution and mitochondrial of offspring. Following hypothesized mating events, we present empirical alignment of Φ_ST_ estimates across markers along the Z‐chromosome and Chromosome‐1 between genetically vetted American black ducks and western mallards, along with each hybrid class (Supporting Information Table S1). Note the clear decrease in outlier regions with each subsequent backcross until F3 (or second generation backcrosses), which are genetically identical to pure American black ducks. Moreover, the hypothesized mating events demonstrate how pure black ducks can retain Old World (OW) A haplotypes irrespective of the nuclear genome (i.e., mitochondrial capture)

Among markers, mitochondrial DNA (mtDNA) has been extensively used to infer gene flow among ducks (Ankney et al., 1986; Avise et al., 1990; Lavretsky, Hernández Baños et al., 2014). However, we find that many mallards and black ducks that are genomically “pure,” possess an A haplotype (Figure 2). Specifically, backcrossing can lead to mitochondrial capture, in which the OW A mtDNA haplotype is retained in a backcrossed lineage that is otherwise indistinguishable from their parental population at their nuclear genome (Figure 8). For example, two black duck samples from the Mississippi flyway carry OW A haplotypes despite having ≥98% genetic assignment to the black duck population (Figure 2). This suggests that introgressed mtDNA can persist longer than introgressed nuclear variants, and in turn suggests overestimated rates of hybridization that need to be carefully considered. Such a scenario may explain why a proportion of all five New World mallard‐like taxa carry the OW A haplotype despite being genetically “pure” (Bonnet, Leblois, Rousset, & Crochet, 2017; Lavretsky, Dacosta et al., 2015; Lavretsky, Hernández Baños et al., 2014).

Next, hypotheses accounting for the presence of Old World (OW) A and New World (NW) B haplogroups within all NW mallard clade species, include bi‐directional introgression and/or ancestral variation (Avise et al., 1990; Johnson & Sorenson, 1999; Lavretsky, McCracken et al., 2014; Livezey, 1991). Under a scenario of wild mallard introgression from birds dispersing eastward from Alaska, we expect a decrease of A haplotypes from west to east. Instead, we find an opposite trend, with the largest proportion of birds on the east coast with A haplotypes and a steady decay in A haplotype frequencies westward (Figure 2). Additionally, the presence of A haplotypes was significantly associated with the autosomal assignment probability to a non‐western mallard group, rather than randomly distributed as expected under a scenario of stochastic ancestral retention. Thus, our study strongly suggests that a great proportion of OW A haplotypes in North America is the result of game‐farm stocking practices. To definitively confirm our results, future work would benefit from sequencing known game‐farm mallard stock that continue to be released into the landscape and augmenting wild populations in Eastern North America. If the original source for North American game‐farm mallards was Eurasian, then we expect ~100% of these to carry the OW A haplotype. In addition, sequencing historical samples may also provide necessary resolution as to the source of the A haplogroup in North America. Specifically, if the OW A haplotype is the result of game‐farm mallard stocking practices, then we expect any mallards in the early part of the 1900s with A haplotype to also be assigned at autosomal markers to game‐farm. We acknowledge that we cannot fully discount the natural introgression of OW haplotypes via wild mallards moving between Eurasia and North America. However, the widespread gene flow of OW A haplotypes from feral mallard stocks may have greatly convoluted the North American gene pool to the point that it is now impossible to conclusively test between competing hypotheses regarding the proximate cause for both mtDNA A and B haplogroups being found in the New World.

### 4.4 Signatures of demographic and selective processes across the genomes of North American black ducks and mallards

We identify several genomic regions that harbor markers with elevated relative divergence and signatures of divergent selection—as determined by BayeScan—between genetically vetted black ducks, western mallards, and non‐western mallards (Figure 6). In an attempt to distinguish between demographic and selective processes across putative outlier, non‐neutral markers, we calculated and compared Tajima's D, nucleotide diversity, and absolute divergence (*d_XY_*). In general, we find evidence for both demographic and selective processes influencing genetic variation and driving observed relative differentiation at outliers. First, as expected, most putative outliers determined using relative differentiation were marked by low nucleotide diversity in at least one of the compared taxa (Supporting Information Figures [Link], [Link], [Link]). Next, our comparison of absolute divergence was complicated due to a strong correlation between nucleotide diversity and absolute divergence. The obtained correlation is most likely due to insufficient time since divergence (~150,000 years before present; Lavretsky, Hernández Baños et al., 2014) that is required to drive differences in these two estimates (Martin et al., 2015). Thus, comparing absolute divergence and relative differentiation as a test between selection and demographic processes acting on the genomes is likely not appropriate in cases of very recent divergence. Nevertheless, comparing putative outlier markers between genetically vetted western mallards and black ducks (Figure 6), we find overall lower nucleotide diversity and negative Tajima's D in western mallards across outlier regions on the Z‐Sex chromosome, Chromosome 1, and Chromosome 2 (Supporting Information Figure S7), which is evidence for, and consistent with divergent selection in western mallards. This is in contrast to findings when comparing non‐western mallards to black ducks (Supporting Information Figure S8) or western mallards (Supporting Information Figure S9), in which we report an overall shift toward positive Tajima's D across outlier and non‐outlier markers. We argue that the shift toward positive Tajima's D in non‐western mallards is most likely the influence of processes associated with domestication (Makino et al., 2018; Randi, 2008). We conclude that divergence between black ducks and mallards, including within mallards, is likely the result of both demographic and selective processes acting in each of them. Future work will greatly benefit from full genome resequencing to fully understand the size and location of outlier regions.

### 4.5 Genomics of hybridization & islands of differentiation

Despite high rates of observed interspecific interactions between black ducks and mallards in the middle part of the 20th century (Ankney et al., 1986; Avise et al., 1990; Heusmann, 1974; Mank et al., 2004; McAuley, Clugston, & Longcore, 1998; Merendino & Ankney, 1994; Merendino, Ankney, & Dennis, 1993), our results do not support the eastern hybrid swarm hypothesis or predict the subsequent genetic extinction of the black duck (Rhymer, 2006). ADMIXTURE analysis of our dataset suggests a minimum ~25% hybridization rate between mallards and black ducks (Table 1), which is substantially higher than rates between mallards and either Mexican ducks (~2%; Lavretsky, Dacosta et al., 2015) or mottled ducks (~5%; Ford, Selman, & Taylor, 2017; Peters et al., 2016). Sustaining such a high rate of hybridization requires special attention as species loss via genetic swamping is highly probable. However, despite these rates, we find that pure black ducks remain on the landscape. Thus, while hybridization may be quite prevalent, such acts may simply be wasted reproductive effort (Leonard, Echegaray, Randi, & Vilà, 2013; Quilodrán, Austerlitz, Currat, & Montoya‐Burgos, 2018) as gene flow via backcrossing appears to be somehow limited. We predict that hybrids are somehow maladaptive as compared to their parentals, and perhaps assortative mating based on plumage or other characteristics by parentals maintains lower levels of actual gene flow between mallards and black ducks. Future work will greatly benefit from utilizing sequence capture techniques to analyze hybridization rates across historical black duck and mallard samples, and determine whether rates have remained the same over time (support for divergence‐with‐gene flow), are decreasing from some previous maximum (support for historical secondary contact), or are continuing to increase (support for recent and ongoing secondary contact).

Alternative to assortative mating limiting gene flow, we hypothesize that the possibility to reestablish the parental lineage through backcrossing in only a few generations may be playing an important role in maintaining black ducks in the face of relative high rates of hybridization. First, the genomes of black ducks and mallards lack any identifiable fixed differences (Figures 4 and 5), with only statistical differences at outlier and ≤1% differences in remaining regions. In fact, using genetically vetted black ducks and western mallards, we demarcated several prominent regions of clustered markers consistent with divergent selection on the Z‐sex chromosome, as well as several autosomal chromosomes (Figure 6). To test for the replacement rate of outlier regions during backcrossing, we compared genetically “pure” black ducks or western mallards to ducks within each hybrid class (Figure 7). We define the outlier replacement rate as the generations of backcrossing required until the region of interest is no longer an outlier between a backcrossed individual and pure parental, and thus providing a relative rate of gene flow (Figure 8). First, outlier comparisons suggest that hybrids backcrossing into black ducks or mallards become genetically indistinguishable from their “pure” parental population within one (F2) or two (F3) generations of backcrossing, respectively (Figure 7) and corresponding to ADMIXTURE simulations (Figure 2) and previous breeding experiments (Kirby et al., 2004). More specifically, outlier regions on the Z‐chromosome and Chromosome‐1 either steadily decayed or increased in overall differentiation when comparing each black duck‐backcrossed generation to either parental black ducks or western mallards, respectively (Figure 7). In fact, Z‐chromosome and Chromosome 1 outlier regions show the near expected 50% decrease in Φ_ST_estimates when comparing F1‐hybrids to pure mallards or black ducks (Figures 6 and 7). The 50% change in Φ_ST_estimates suggest that these regions recombine, with hybrids harboring both mallard and black duck variation. Conversely, regardless of hybrid class, outlier regions on chromosome 2, 5, & 21 maintained their original high differentiation regardless of comparison with western mallards and were entirely absent when compared to black ducks within one generation of backcrossing (Figure 7). Together, these results suggest that latter regions are less likely, if at all able to introgress between the two species. In contrast, the steady decay or increase of outlier regions on the Z‐chromosome and Chromosome 1 suggests that these regions require a minimum of two backcrosses to restore the parental genotype. We acknowledge that ddRAD markers represent ~0.04% of the genome, making it highly probable that many important genomic regions have been missed. Importantly, with the ability to genetically vet wild samples as pure, F1, or generational backcrosses, now permits for the use of wild samples to further test how various genomic regions recombine and interact in intermediate forms.

We find that mallards and black ducks may be best explained by few key genomic regions that are under alternative evolutionary forces (Figure 6) Specifically, we hypothesize that outside genomic regions that are essential to develop into a black duck or mallard, much of the genome codes for basic “*Anas*” functionality, and likely free to move during gene flow events (Figure 8). Thus, while the rate of hybridization and hybrids may be prevalent, the detection of outlier regions suggests these are less likely to move between species, otherwise we would expect these to have been lost. However, whether demarcated outlier regions are on the path to being lost due to gene flow remains unknown. If gene flow between mallards and black ducks has amalgamated much of their genome, while reinforcing these outlier regions, then using historical museum specimens to comparing pre‐ versus post‐secondary contact groups should identify increased differences at said outlier regions and decreased differences across the remaining genome. Alternatively, if black ducks and mallards have been a case of divergence‐with‐gene flow, then comparing pre‐ versus post‐secondary contact should yield similar or increased divergence at outlier regions, while the remaining genome being at similar low levels of divergence.

### 4.6 Conservation implications

Based on ADMIXTURE analyses, we determined that at least ~20% of all phenotypically identified mallards and black ducks were incorrect and possessed hybrid ancestry. Furthermore, only ~60% of phenotypically identified hybrids were true hybrids with ~12% and ~25% of remaining samples being actually “pure” mallards or black ducks, respectively (Figure 2, Table 1; Supporting Information Table S1). Our “error rates” are similar to those reported between mallards and Florida Mottled ducks before a genetically vetted field key was developed (Bielefeld et al., 2016). With the exception of western mallards, all Mississippi and Atlantic flyway mallards, black ducks, and hybrids were sampled from the 2010 U.S. Fish and Wildlife Services' flyway Waterfowl Wingbee meetings. At the meeting, sex, age, and species/status assignments are based on previously characterized wing plumages believed to be diagnostic between black ducks, mallards, and their hybrids (see Kirby et al., 2000); however, the diagnosability of these traits, particularly for hybrids, had never been genetically vetted. For example, the presence of an anterior and/or posterior white‐wing bar across the speculum is currently a primary character used to identify hybrids between mallards and black ducks. However, a recent study found the same character thought to be indicative of hybrids between mallards and mottled ducks in 10% of genetically “pure” mottled ducks (Bielefeld et al., 2016). Thus, for scenarios of recent divergence in which ancestral characters may be maintained across lineages, it is critical to genetically vet phenotypic characters to confirm their diagnosability. While outside the scope of this paper, using established genetic assignments, it is now possible to test and identify which species‐cohort require(s) phenotypic re‐evaluation, including identifying those characters that are truly diagnostic of hybrids. Without genetically vetting traits, the misidentification of samples can bias population estimates, hybrid identification, including over/underestimation of the impact of hybridization, behavioral studies, and any other study requiring proper species identification. We acknowledge that although breeding experiments would be ideal to validate simulations, our results closely correspond to previous breeding experiments (Kirby et al., 2004). Thus, we believe that outlined methods provide researchers a means to establish the number and generational hybrid classes in their datasets, allowing for a more accurate assessment of hybridization and introgression in wild populations.

## CONFLICT OF INTEREST

PL, TJ, & KGM have no financial interest to report.

## AUTHOR CONTRIBUTIONS

PL conceptualized, collected, and analyzed data. KGM conceptualized and supported data acquisition. TJ conceptualized and analyzed data. PL, TJ, & KGM equally contributed to the writing of this manuscript.

## Data Availability

Mitochondrial DNA sequences: GenBank accessions MK425222‐MK425495. Illumina ddRAD‐Seq Reads: NCBI's Sequence Read Archive data PRJNA516035: BioSample. Accession Numbers SAMN10781288‐ SAMN10781577. Other Data Files (FASTA, ADMIXTURE/Simulations, PCA, Haplotype Block/ANCESTRY_HHM Input Files): Dryad accession https://doi.org/10.5061/dryad.060b8n9.
