# Supplementary figures and images for: Identifying hybrids & the genomics of hybridization: Mallards & American black ducks of Eastern North America
Source: Ecol Evol. 2019 Feb 27;9(6):3470–90. doi: 10.1002/ece3.4981 (PMC6434578; doi:10.1002/ece3.4981)

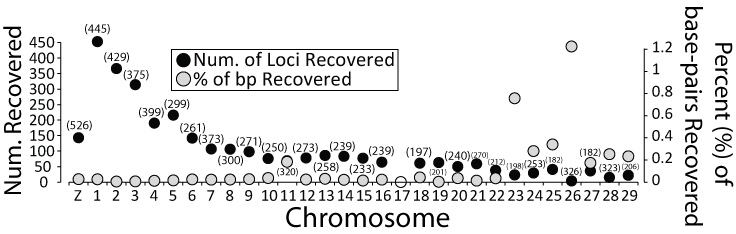

Supplement: Supplementary file 1 [file ECE3-9-3470-s001.png]

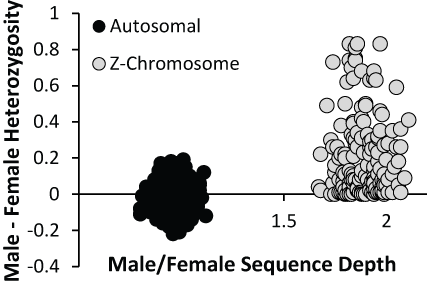

Supplement: Supplementary file 2 [file ECE3-9-3470-s003.png]

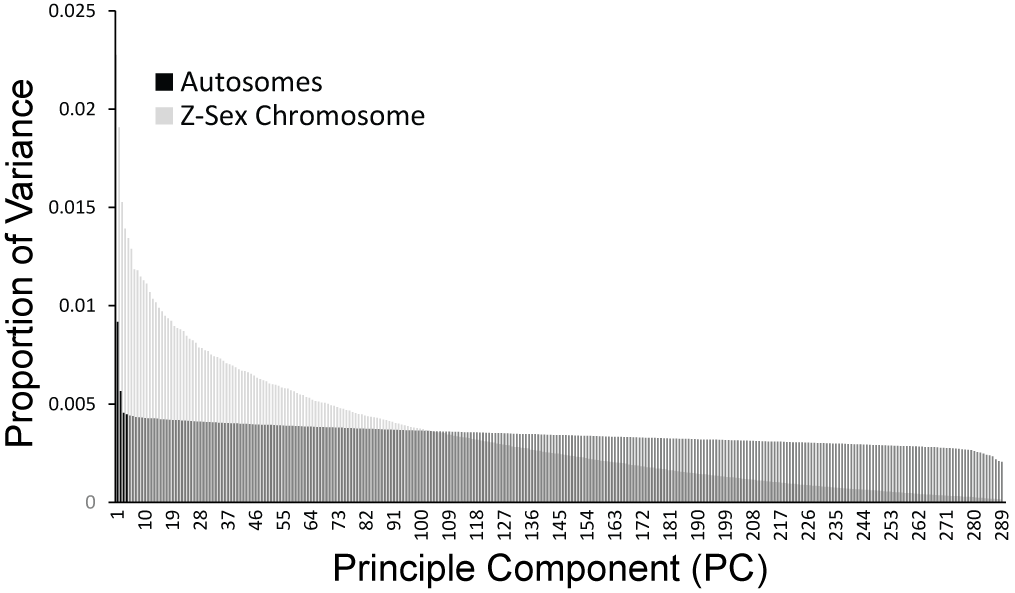

Supplement: Supplementary file 3 [file ECE3-9-3470-s004.png]

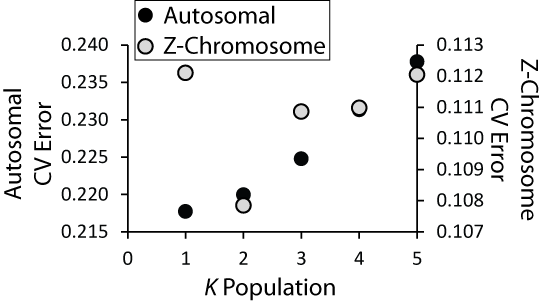

Supplement: Supplementary file 4 [file ECE3-9-3470-s005.png]

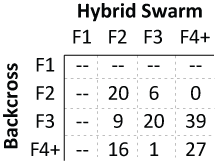

Supplement: Supplementary file 6 [file ECE3-9-3470-s007.png]

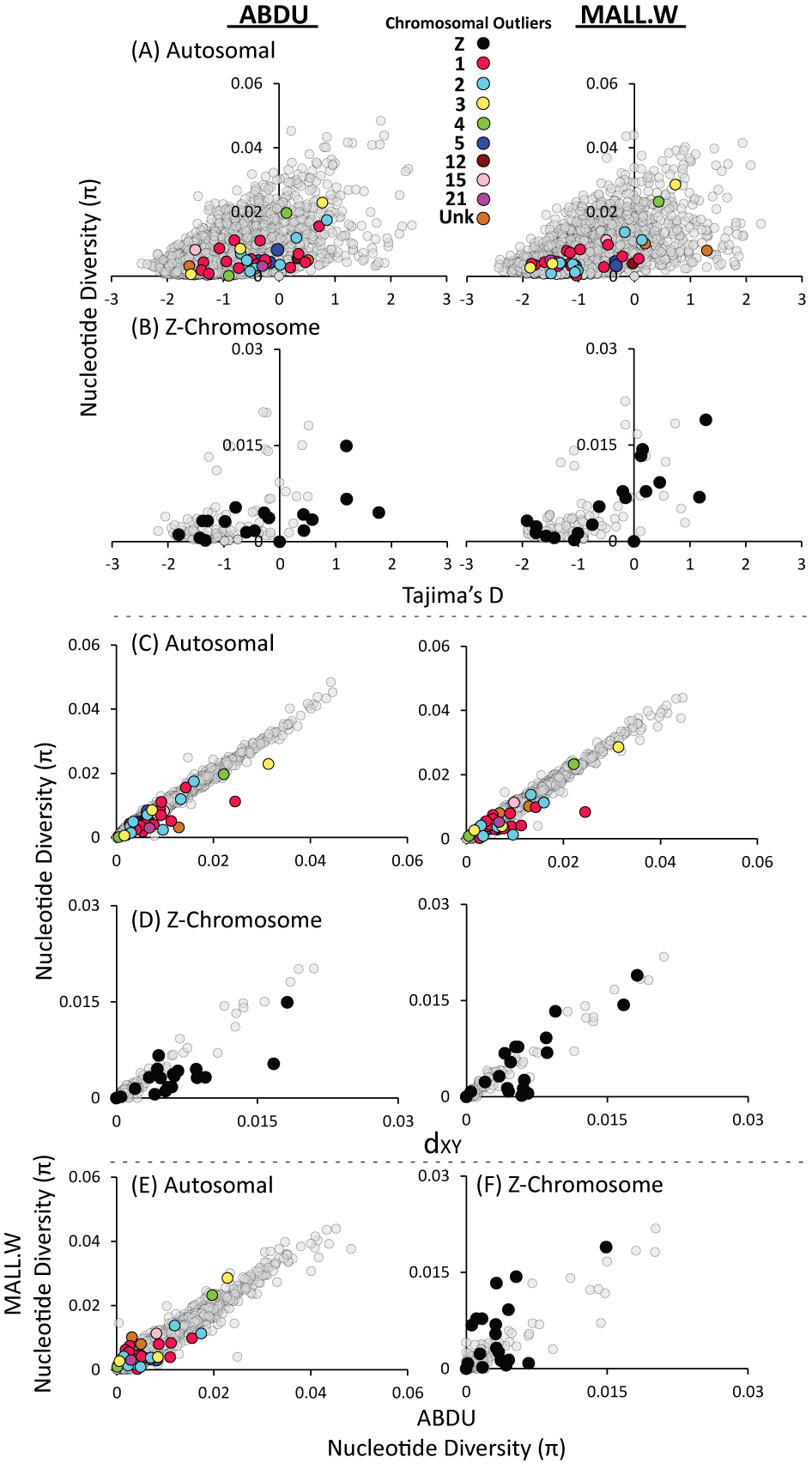

Supplement: Supplementary file 7 [file ECE3-9-3470-s008.png]

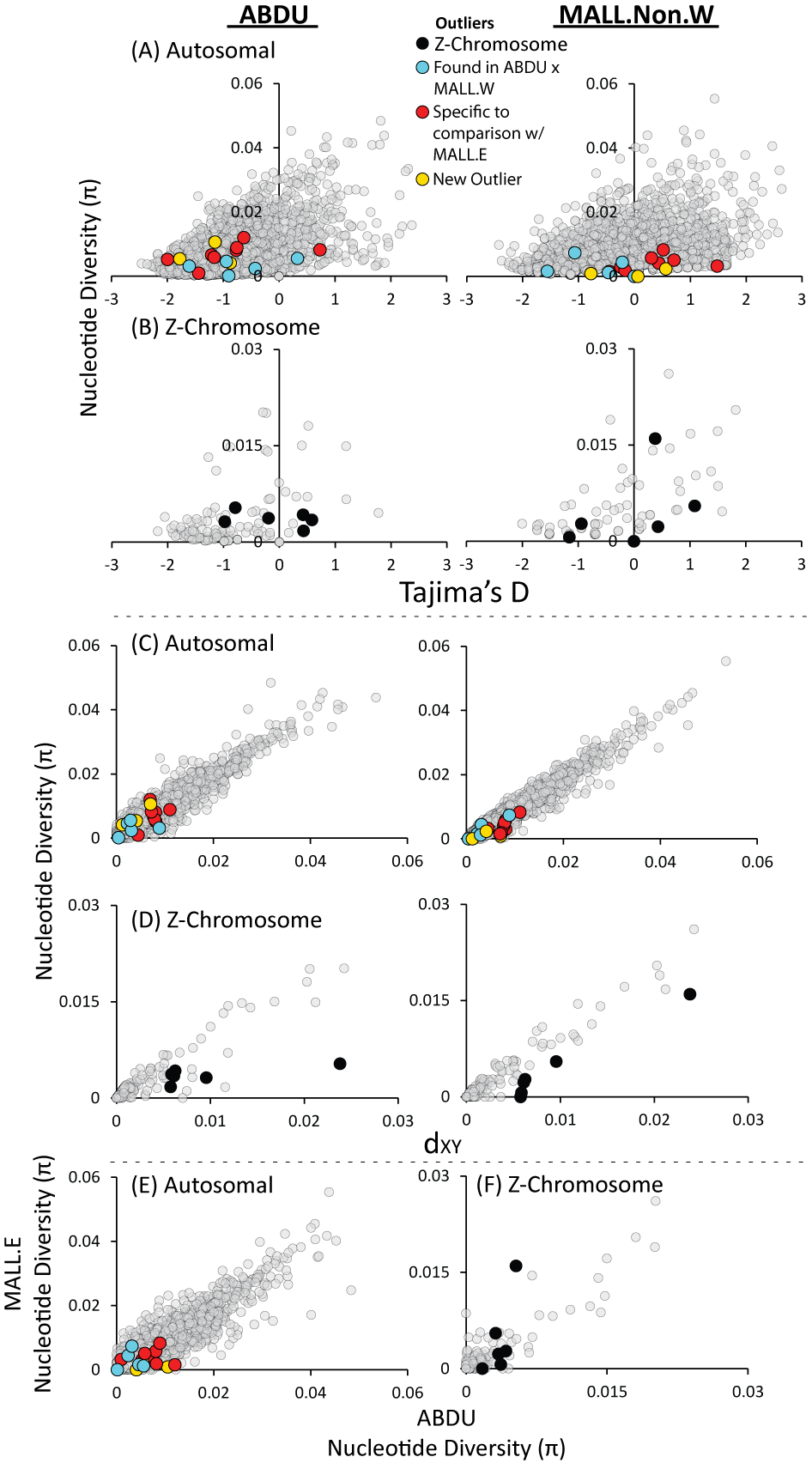

Supplement: Supplementary file 8 [file ECE3-9-3470-s009.png]

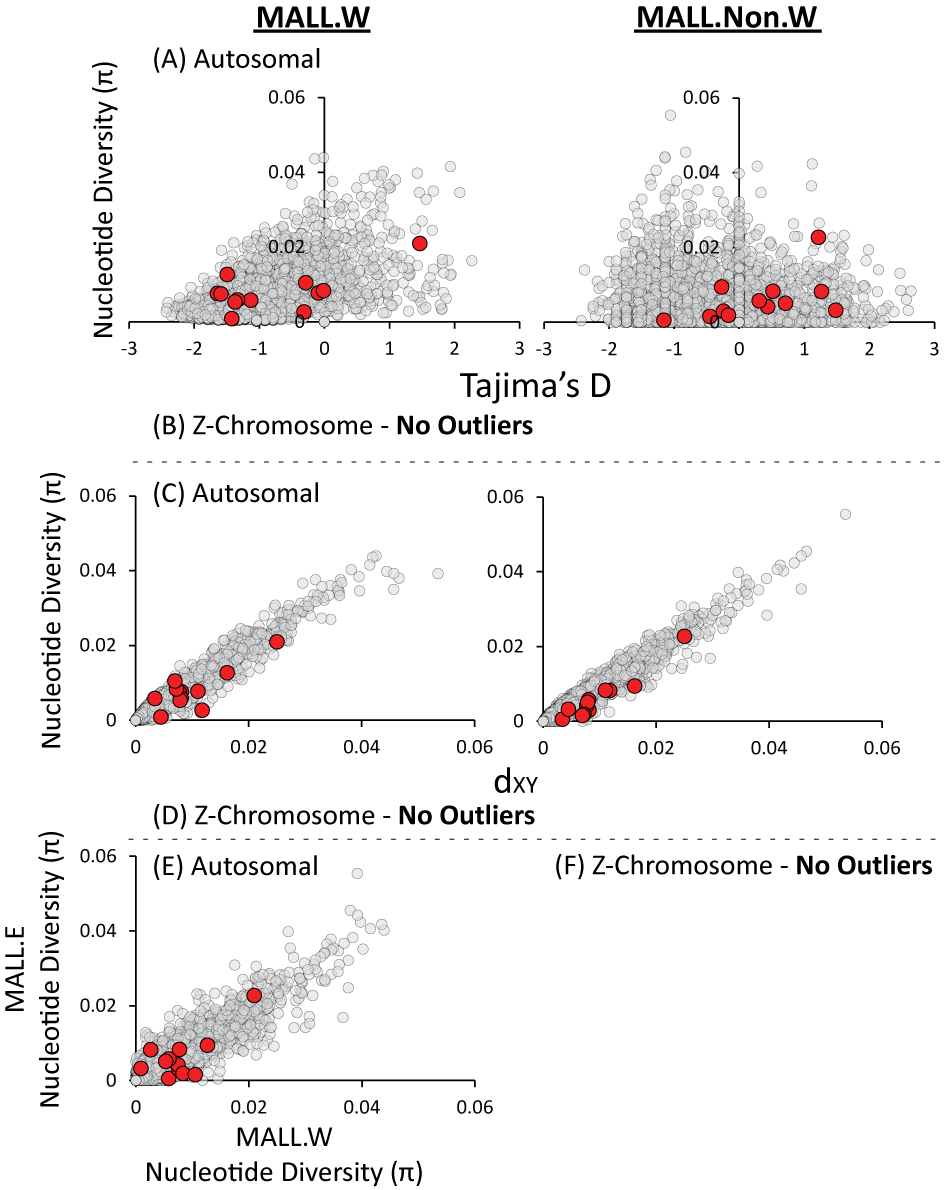

Supplement: Supplementary file 9 [file ECE3-9-3470-s010.png]
