# Supplementary material for: Identifying hybrids & the genomics of hybridization: Mallards & American black ducks of Eastern North America
Source: Ecol Evol. 2019 Feb 27;9(6):3470–90. doi: 10.1002/ece3.4981 (PMC6434578; doi:10.1002/ece3.4981)
Supplement: Supplementary file 5 [file ECE3-9-3470-s006.pdf]

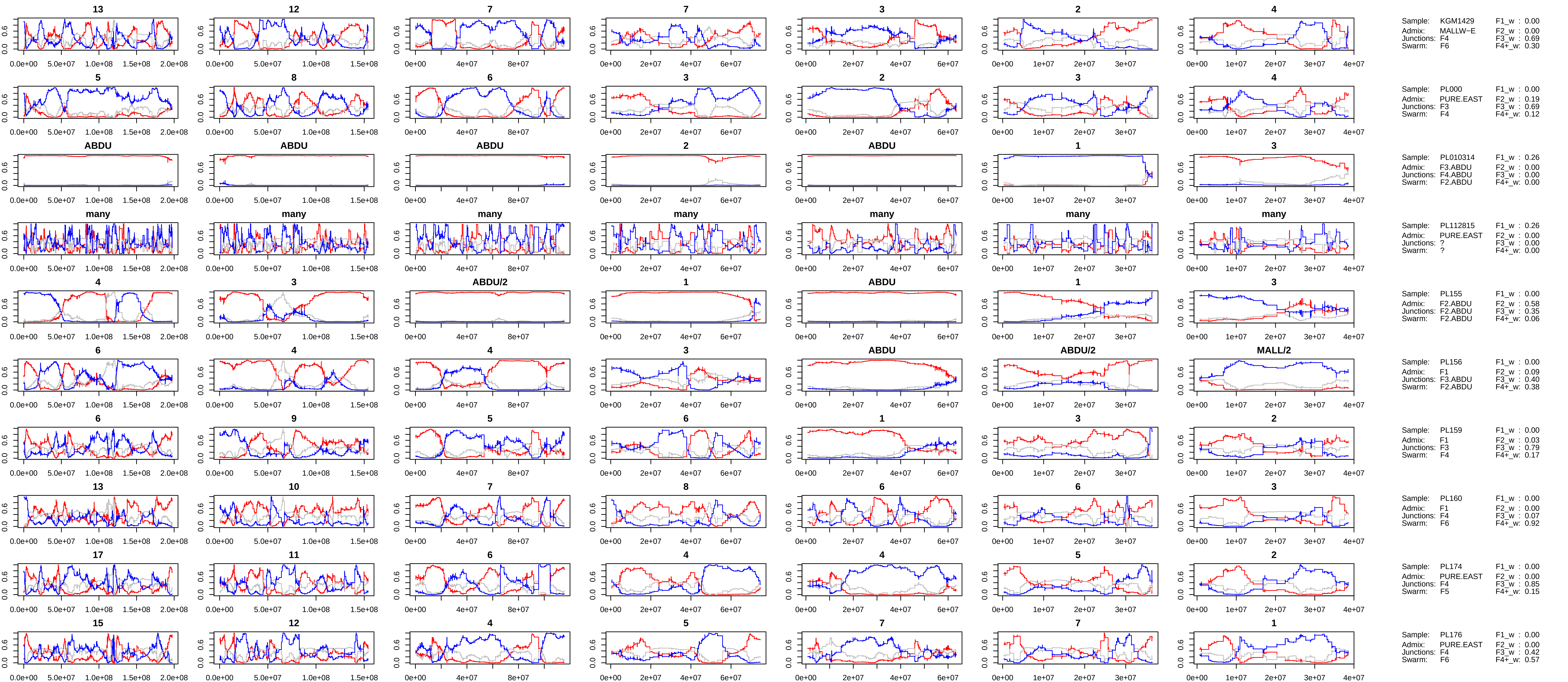
13 12 7 7 3 2 4
Sample: KGM1429 F1_w : 0.00
Admix: MALLW−E F2_w : 0.00
Junctions: F4 F3_w : 0.69
Swarm: F6 F4+_w: 0.30
5 8 6 3 2 3 4
Sample: PL000 F1_w : 0.00
Admix: PURE.EAST F2_w : 0.19
Junctions: F3 F3_w : 0.69
Swarm: F4 F4+_w: 0.12
ABDU ABDU ABDU 2 ABDU 1 3
Sample: PL010314 F1_w : 0.26
Admix: F3.ABDU F2_w : 0.00
Junctions: F4.ABDU F3_w : 0.00
Swarm: F2.ABDU F4+_w: 0.00
many many many many many many many
Sample: PL112815 F1_w : 0.26
Admix: PURE.EAST F2_w : 0.00
Junctions: ? F3_w : 0.00
Swarm: ? F4+_w: 0.00
4 3 ABDU/2 1 ABDU 1 3
Sample: PL155 F1_w : 0.00
Admix: F2.ABDU F2_w : 0.58
Junctions: F2.ABDU F3_w : 0.35
Swarm: F2.ABDU F4+_w: 0.06
6 4 4 3 ABDU ABDU/2 MALL/2
Sample: PL156 F1_w : 0.00
Admix: F1 F2_w : 0.09
Junctions: F3.ABDU F3_w : 0.40
Swarm: F2.ABDU F4+_w: 0.38
6 9 5 6 1 3 2
Sample: PL159 F1_w : 0.00
Admix: F1 F2_w : 0.03
Junctions: F3 F3_w : 0.79
Swarm: F4 F4+_w: 0.17
13 10 7 8 6 6 3
Sample: PL160 F1_w : 0.00
Admix: F1 F2_w : 0.00
Junctions: F4 F3_w : 0.07
Swarm: F6 F4+_w: 0.92
17 11 6 4 4 5 2
Sample: PL174 F1_w : 0.00
Admix: PURE.EAST F2_w : 0.00
Junctions: F4 F3_w : 0.85
Swarm: F5 F4+_w: 0.15
15 12 4 5 7 7 1
Sample: PL176 F1_w : 0.00
Admix: PURE.EAST F2_w : 0.00
Junctions: F4 F3_w : 0.42
Swarm: F6 F4+_w: 0.57

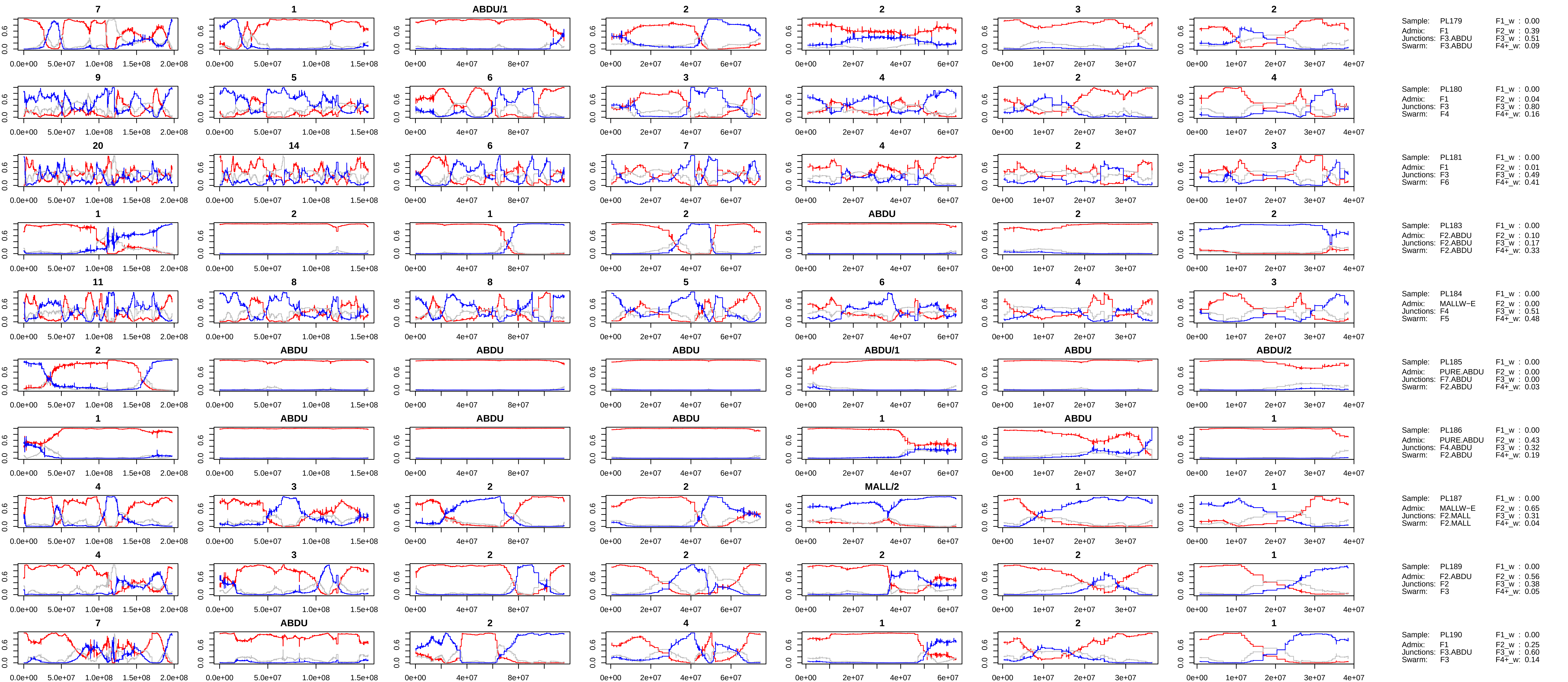

7
1
ABDU/1
2
2
3
2
Sample: PL179
Admix: F1
Junctions: F3.ABDU
Swarm: F3.ABDU
F1_w : 0.00
F2_w : 0.39
F3_w : 0.51
F4+_w: 0.09
9
5
6
3
4
2
4
Sample: PL180
Admix: F1
Junctions: F3
Swarm: F4
F1_w : 0.00
F2_w : 0.04
F3_w : 0.80
F4+_w: 0.16
20
14
6
7
4
2
3
Sample: PL181
Admix: F1
Junctions: F3
Swarm: F6
F1_w : 0.00
F2_w : 0.01
F3_w : 0.49
F4+_w: 0.41
1
2
1
2
ABDU
2
2
Sample: PL183
Admix: F2.ABDU
Junctions: F2.ABDU
Swarm: F2.ABDU
F1_w : 0.00
F2_w : 0.10
F3_w : 0.17
F4+_w: 0.33
11
8
8
5
6
4
3
Sample: PL184
Admix: MALLW−E
Junctions: F4
Swarm: F5
F1_w : 0.00
F2_w : 0.00
F3_w : 0.51
F4+_w: 0.48
2
ABDU
ABDU
ABDU
ABDU/1
ABDU
ABDU/2
Sample: PL185
Admix: PURE.ABDU
Junctions: F7.ABDU
Swarm: F2.ABDU
F1_w : 0.00
F2_w : 0.00
F3_w : 0.00
F4+_w: 0.03
1
ABDU
ABDU
ABDU
1
ABDU
1
Sample: PL186
Admix: PURE.ABDU
Junctions: F4.ABDU
Swarm: F2.ABDU
F1_w : 0.00
F2_w : 0.43
F3_w : 0.32
F4+_w: 0.19
4
3
2
2
MALL/2
1
1
Sample: PL187
Admix: MALLW−E
Junctions: F2.MALL
Swarm: F2.MALL
F1_w : 0.00
F2_w : 0.65
F3_w : 0.31
F4+_w: 0.04
4
3
2
2
2
2
1
Sample: PL189
Admix: F2.ABDU
Junctions: F2
Swarm: F3
F1_w : 0.00
F2_w : 0.56
F3_w : 0.38
F4+_w: 0.05
7
ABDU
2
4
1
2
1
Sample: PL190
Admix: F1
Junctions: F3.ABDU
Swarm: F3
F1_w : 0.00
F2_w : 0.25
F3_w : 0.60
F4+_w: 0.14

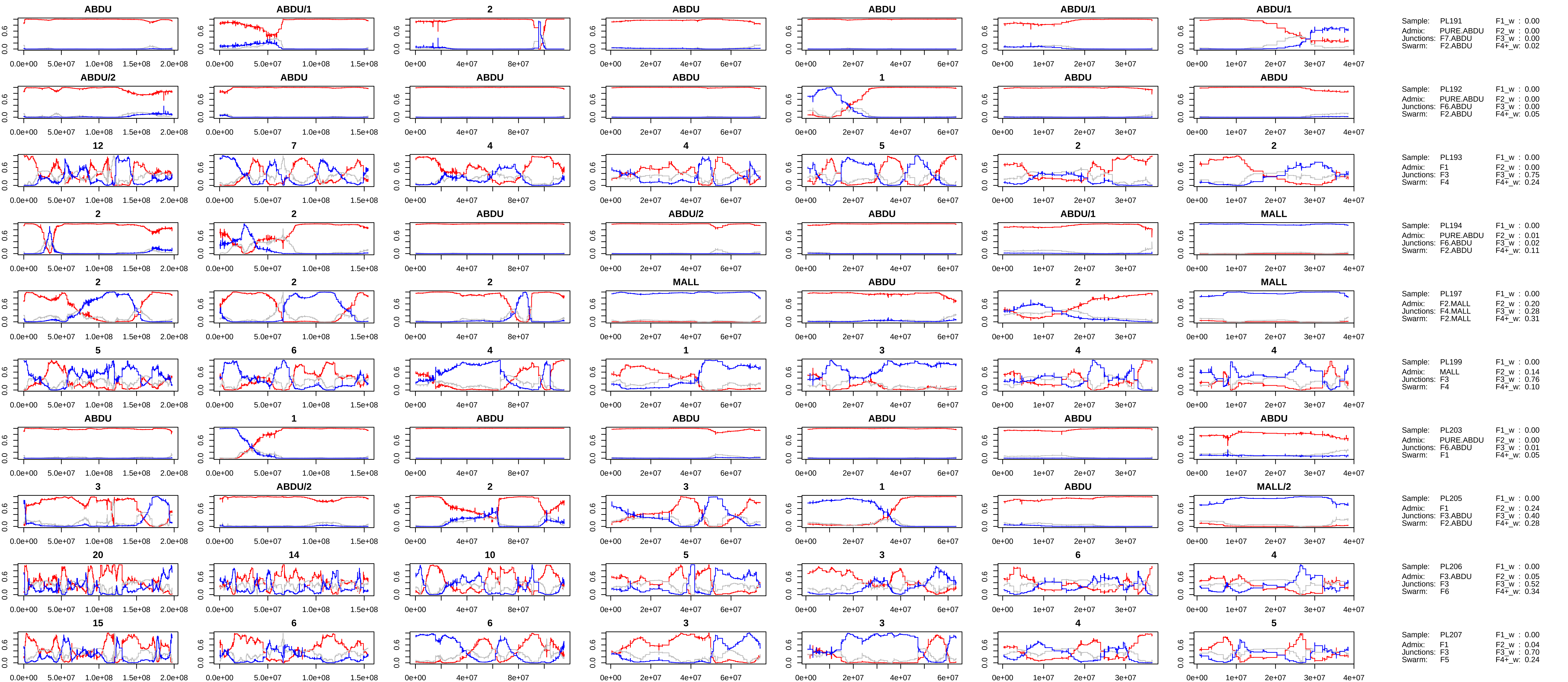
ABDU ABDU/1 2 ABDU ABDU ABDU/1 ABDU/1
Sample: PL191 F1_w : 0.00
Admix: PURE.ABDU F2_w : 0.00
Junctions: F7.ABDU F3_w : 0.00
Swarm: F2.ABDU F4+_w: 0.02
ABDU/2 ABDU ABDU ABDU 1 ABDU ABDU
Sample: PL192 F1_w : 0.00
Admix: PURE.ABDU F2_w : 0.00
Junctions: F6.ABDU F3_w : 0.00
Swarm: F2.ABDU F4+_w: 0.05
12 7 4 4 5 2 2
Sample: PL193 F1_w : 0.00
Admix: F1 F2_w : 0.00
Junctions: F3 F3_w : 0.75
Swarm: F4 F4+_w: 0.24
2 2 ABDU ABDU/2 ABDU ABDU/1 MALL
Sample: PL194 F1_w : 0.00
Admix: PURE.ABDU F2_w : 0.01
Junctions: F6.ABDU F3_w : 0.02
Swarm: F2.ABDU F4+_w: 0.11
2 2 2 MALL ABDU 2 MALL
Sample: PL197 F1_w : 0.00
Admix: F2.MALL F2_w : 0.20
Junctions: F4.MALL F3_w : 0.28
Swarm: F2.MALL F4+_w: 0.31
5 6 4 1 3 4 4
Sample: PL199 F1_w : 0.00
Admix: MALL F2_w : 0.14
Junctions: F3 F3_w : 0.76
Swarm: F4 F4+_w: 0.10
ABDU 1 ABDU ABDU ABDU ABDU ABDU
Sample: PL203 F1_w : 0.00
Admix: PURE.ABDU F2_w : 0.00
Junctions: F6.ABDU F3_w : 0.01
Swarm: F1 F4+_w: 0.05
3 ABDU/2 2 3 1 ABDU MALL/2
Sample: PL205 F1_w : 0.00
Admix: F1 F2_w : 0.24
Junctions: F3.ABDU F3_w : 0.40
Swarm: F2.ABDU F4+_w: 0.28
20 14 10 5 3 6 4
Sample: PL206 F1_w : 0.00
Admix: F3.ABDU F2_w : 0.05
Junctions: F3 F3_w : 0.52
Swarm: F6 F4+_w: 0.34
15 6 6 3 3 4 5
Sample: PL207 F1_w : 0.00
Admix: F1 F2_w : 0.04
Junctions: F3 F3_w : 0.70
Swarm: F5 F4+_w: 0.24

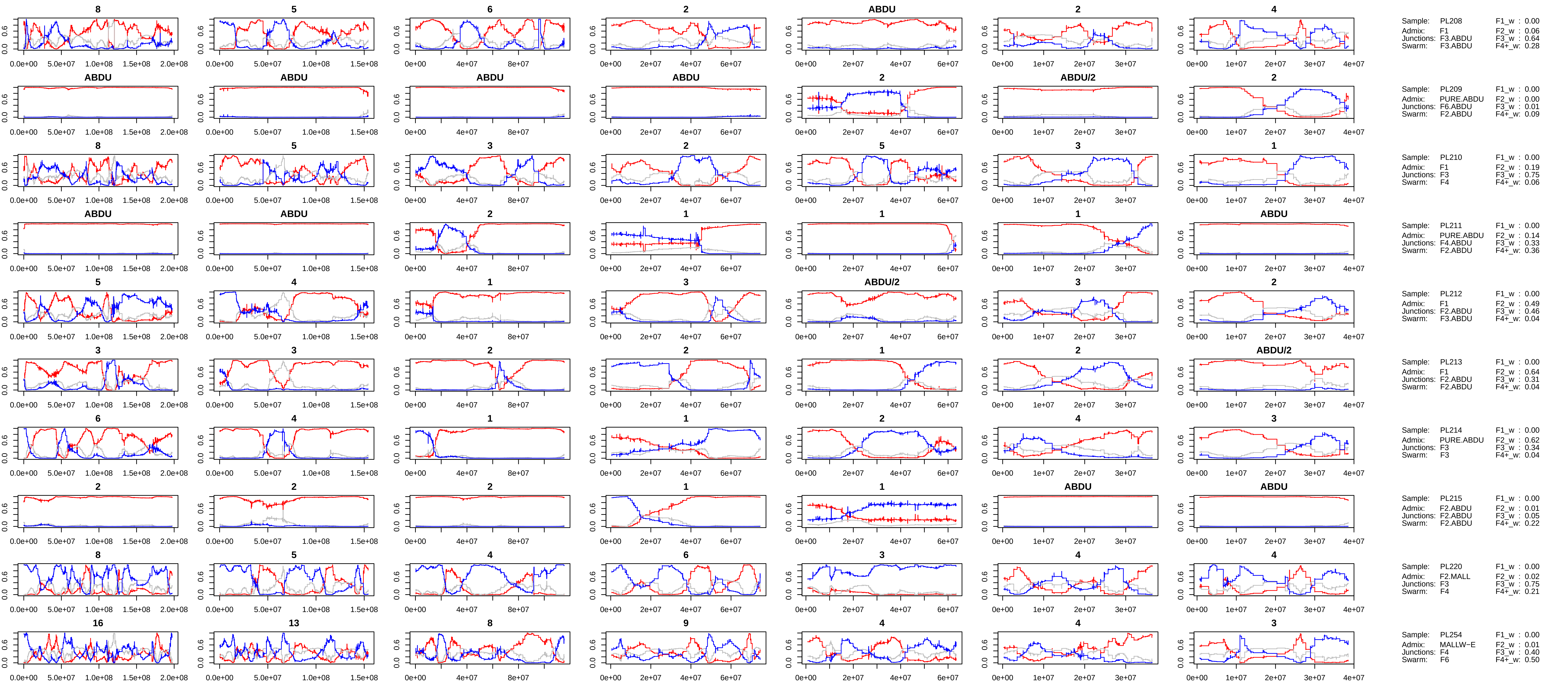
8 5 6 2 ABDU 2 4
Sample: PL208 F1_w : 0.00
Admix: F1 F2_w : 0.06
Junctions: F3.ABDU F3_w : 0.64
Swarm: F3.ABDU F4+_w: 0.28
ABDU ABDU ABDU ABDU 2 ABDU/2 2
Sample: PL209 F1_w : 0.00
Admix: PURE.ABDU F2_w : 0.00
Junctions: F6.ABDU F3_w : 0.01
Swarm: F2.ABDU F4+_w: 0.09
8 5 3 2 5 3 1
Sample: PL210 F1_w : 0.00
Admix: F1 F2_w : 0.19
Junctions: F3 F3_w : 0.75
Swarm: F4 F4+_w: 0.06
ABDU ABDU 2 1 1 1 ABDU
Sample: PL211 F1_w : 0.00
Admix: PURE.ABDU F2_w : 0.14
Junctions: F4.ABDU F3_w : 0.33
Swarm: F2.ABDU F4+_w: 0.36
5 4 1 3 ABDU/2 3 2
Sample: PL212 F1_w : 0.00
Admix: F1 F2_w : 0.49
Junctions: F2.ABDU F3_w : 0.46
Swarm: F3.ABDU F4+_w: 0.04
3 3 2 2 1 2 ABDU/2
Sample: PL213 F1_w : 0.00
Admix: F1 F2_w : 0.64
Junctions: F2.ABDU F3_w : 0.31
Swarm: F2.ABDU F4+_w: 0.04
6 4 1 1 2 4 3
Sample: PL214 F1_w : 0.00
Admix: PURE.ABDU F2_w : 0.62
Junctions: F3 F3_w : 0.34
Swarm: F3 F4+_w: 0.04
2 2 2 1 1 ABDU ABDU
Sample: PL215 F1_w : 0.00
Admix: F2.ABDU F2_w : 0.01
Junctions: F2.ABDU F3_w : 0.05
Swarm: F2.ABDU F4+_w: 0.22
8 5 4 6 3 4 4
Sample: PL220 F1_w : 0.00
Admix: F2.MALL F2_w : 0.02
Junctions: F3 F3_w : 0.75
Swarm: F4 F4+_w: 0.21
16 13 8 9 4 4 3
Sample: PL254 F1_w : 0.00
Admix: MALLW−E F2_w : 0.01
Junctions: F4 F3_w : 0.40
Swarm: F6 F4+_w: 0.50

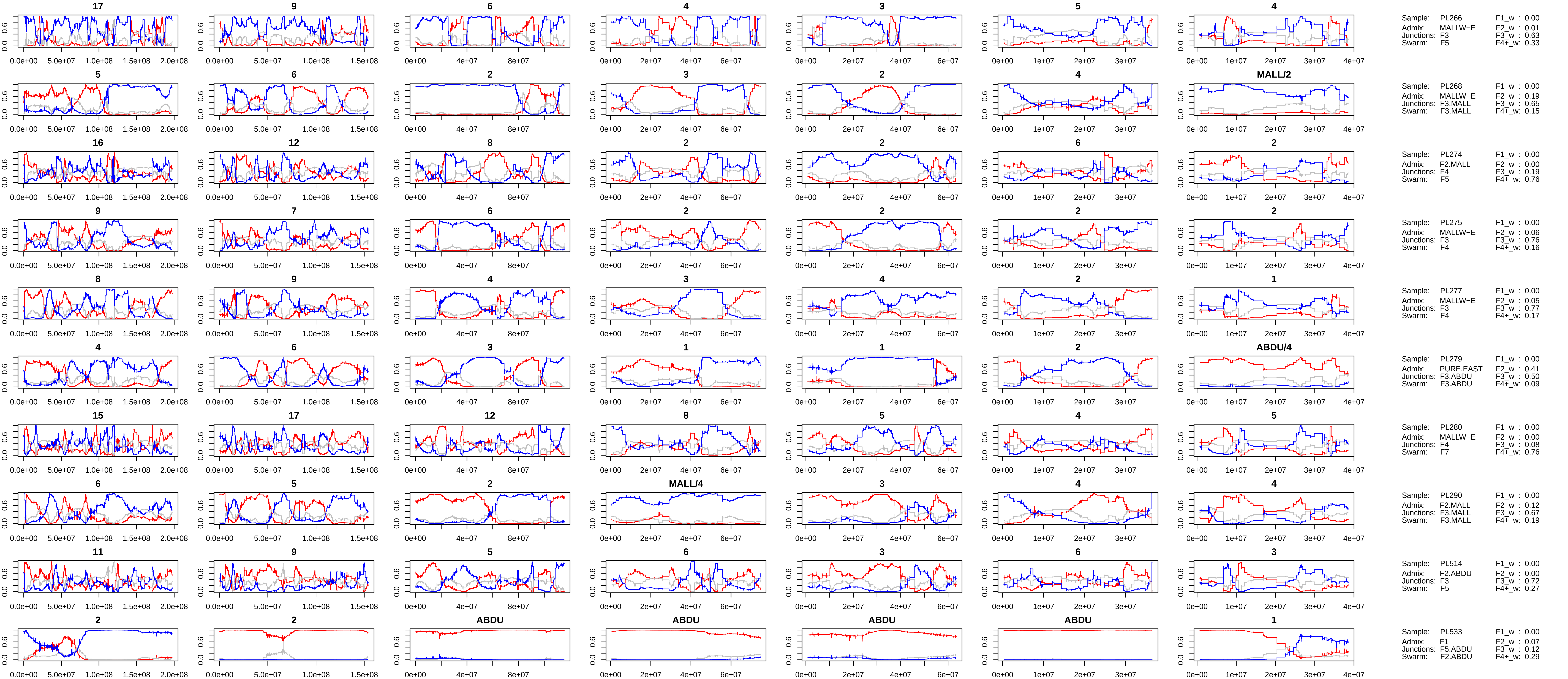
17 9 6 4 3 5 4
Sample: PL266 F1_w : 0.00
Admix: MALLW−E F2_w : 0.01
Junctions: F3 F3_w : 0.63
Swarm: F5 F4+_w: 0.33
5 6 2 3 2 4 MALL/2
Sample: PL268 F1_w : 0.00
Admix: MALLW−E F2_w : 0.19
Junctions: F3.MALL F3_w : 0.65
Swarm: F3.MALL F4+_w: 0.15
16 12 8 2 2 6 2
Sample: PL274 F1_w : 0.00
Admix: F2.MALL F2_w : 0.00
Junctions: F4 F3_w : 0.19
Swarm: F5 F4+_w: 0.76
9 7 6 2 2 2 2
Sample: PL275 F1_w : 0.00
Admix: MALLW−E F2_w : 0.06
Junctions: F3 F3_w : 0.76
Swarm: F4 F4+_w: 0.16
8 9 4 3 4 2 1
Sample: PL277 F1_w : 0.00
Admix: MALLW−E F2_w : 0.05
Junctions: F3 F3_w : 0.77
Swarm: F4 F4+_w: 0.17
4 6 3 1 1 2 ABDU/4
Sample: PL279 F1_w : 0.00
Admix: PURE.EAST F2_w : 0.41
Junctions: F3.ABDU F3_w : 0.50
Swarm: F3.ABDU F4+_w: 0.09
15 17 12 8 5 4 5
Sample: PL280 F1_w : 0.00
Admix: MALLW−E F2_w : 0.00
Junctions: F4 F3_w : 0.08
Swarm: F7 F4+_w: 0.76
6 5 2 MALL/4 3 4 4
Sample: PL290 F1_w : 0.00
Admix: F2.MALL F2_w : 0.12
Junctions: F3.MALL F3_w : 0.67
Swarm: F3.MALL F4+_w: 0.19
11 9 5 6 3 6 3
Sample: PL514 F1_w : 0.00
Admix: F2.ABDU F2_w : 0.00
Junctions: F3 F3_w : 0.72
Swarm: F5 F4+_w: 0.27
2 2 ABDU ABDU ABDU ABDU 1
Sample: PL533 F1_w : 0.00
Admix: F1 F2_w : 0.07
Junctions: F5.ABDU F3_w : 0.12
Swarm: F2.ABDU F4+_w: 0.29

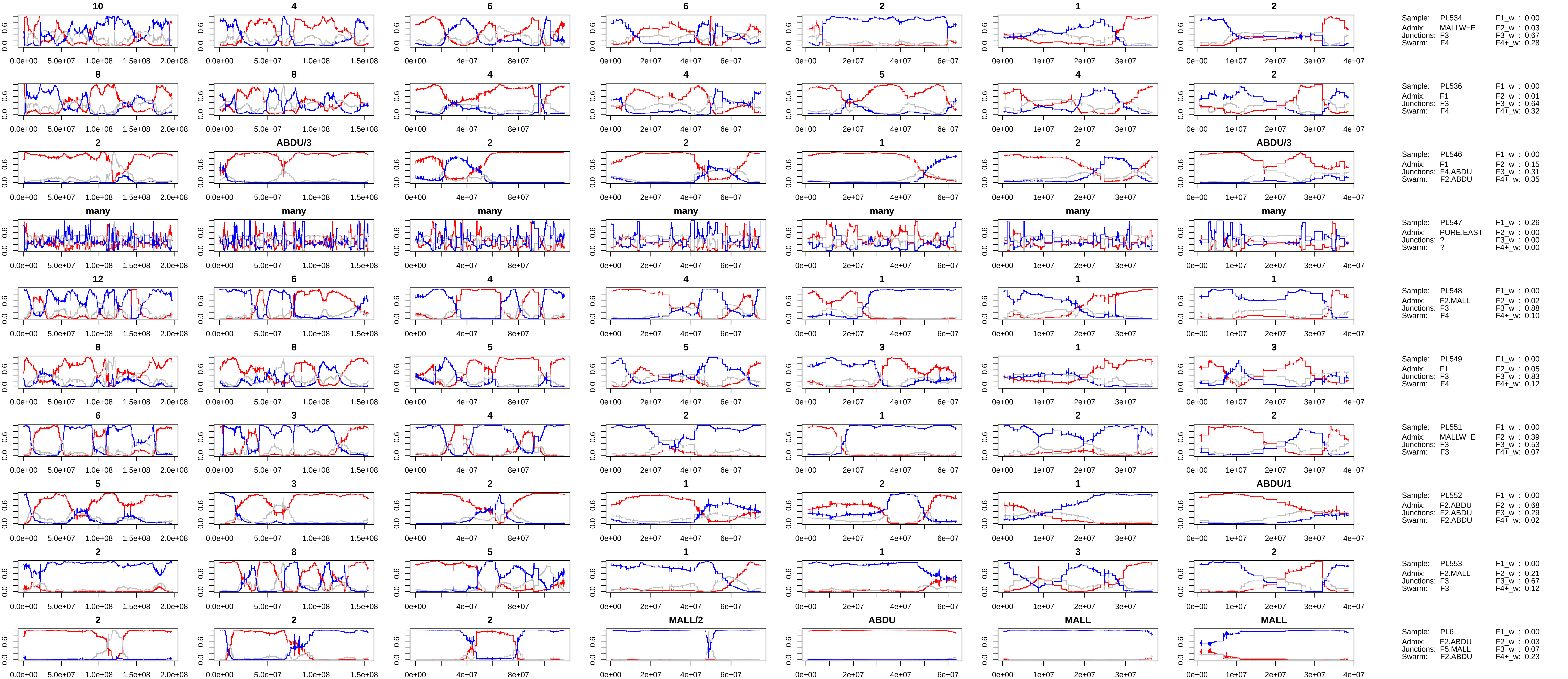

10
4
6
6
2
1
2
Sample: PL534
Admix: MALLW−E
Junctions: F3
Swarm: F4
F1_w : 0.00
F2_w : 0.03
F3_w : 0.67
F4+_w: 0.28
8
8
4
4
5
4
2
Sample: PL536
Admix: F1
Junctions: F3
Swarm: F4
F1_w : 0.00
F2_w : 0.01
F3_w : 0.64
F4+_w: 0.32
2
ABDU/3
2
2
1
2
ABDU/3
Sample: PL546
Admix: F1
Junctions: F4.ABDU
Swarm: F2.ABDU
F1_w : 0.00
F2_w : 0.15
F3_w : 0.31
F4+_w: 0.35
many
many
many
many
many
many
many
Sample: PL547
Admix: PURE.EAST
Junctions: ?
Swarm: ?
F1_w : 0.26
F2_w : 0.00
F3_w : 0.00
F4+_w: 0.00
12
6
4
4
1
1
1
Sample: PL548
Admix: F2.MALL
Junctions: F3
Swarm: F4
F1_w : 0.00
F2_w : 0.02
F3_w : 0.88
F4+_w: 0.10
8
8
5
5
3
1
3
Sample: PL549
Admix: F1
Junctions: F3
Swarm: F4
F1_w : 0.00
F2_w : 0.05
F3_w : 0.83
F4+_w: 0.12
6
3
4
2
1
2
2
Sample: PL551
Admix: MALLW−E
Junctions: F3
Swarm: F3
F1_w : 0.00
F2_w : 0.39
F3_w : 0.53
F4+_w: 0.07
5
3
2
1
2
1
ABDU/1
Sample: PL552
Admix: F2.ABDU
Junctions: F2.ABDU
Swarm: F2.ABDU
F1_w : 0.00
F2_w : 0.68
F3_w : 0.29
F4+_w: 0.02
2
8
5
1
1
3
2
Sample: PL553
Admix: F2.MALL
Junctions: F3
Swarm: F3
F1_w : 0.00
F2_w : 0.21
F3_w : 0.67
F4+_w: 0.12
2
2
2
MALL/2
ABDU
MALL
MALL
Sample: PL6
Admix: F2.ABDU
Junctions: F5.MALL
Swarm: F2.ABDU
F1_w : 0.00
F2_w : 0.03
F3_w : 0.07
F4+_w: 0.23

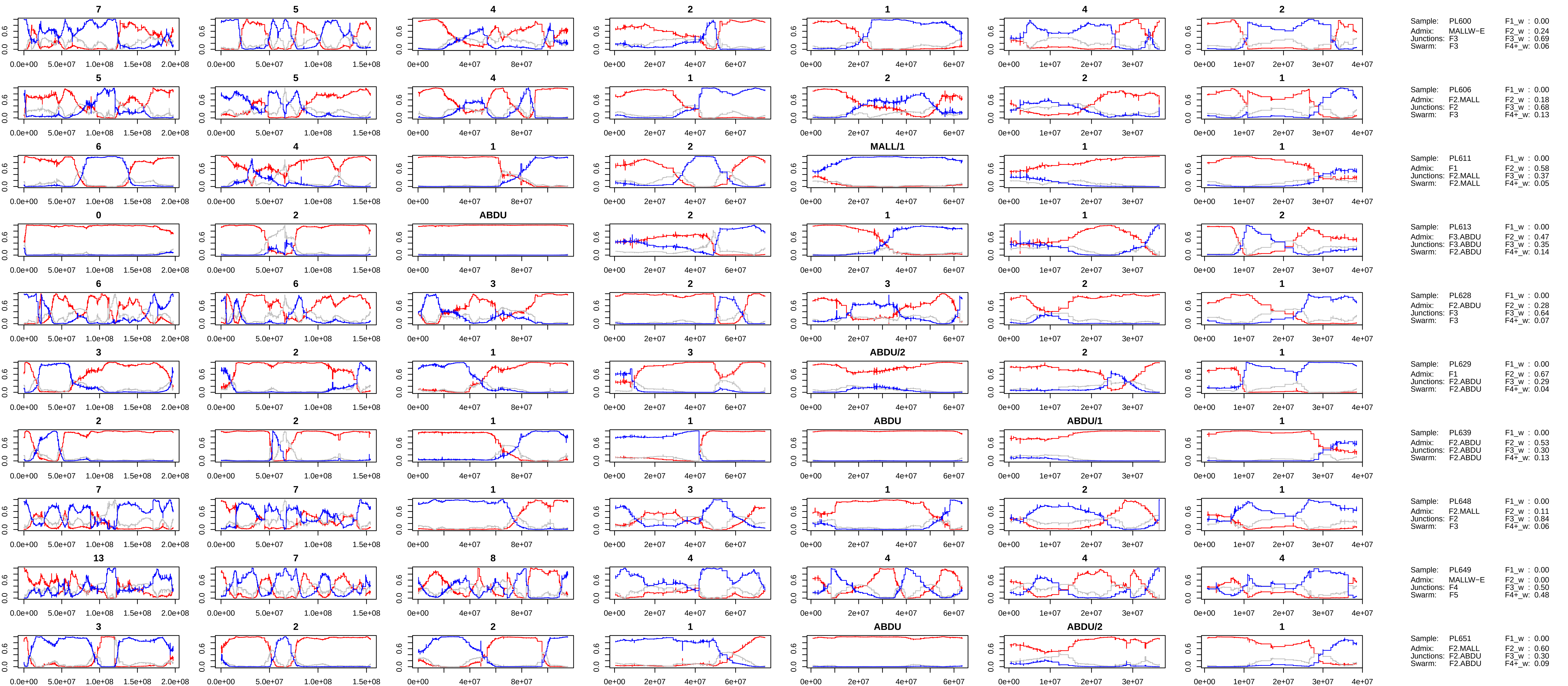
7 5 4 2 1 4 2
Sample: PL600 F1_w : 0.00
Admix: MALLW−E F2_w : 0.24
Junctions: F3 F3_w : 0.69
Swarm: F3 F4+_w: 0.06
5 5 4 1 2 2 1
Sample: PL606 F1_w : 0.00
Admix: F2.MALL F2_w : 0.18
Junctions: F2 F3_w : 0.68
Swarm: F3 F4+_w: 0.13
6 4 1 2 MALL/1 1 1
Sample: PL611 F1_w : 0.00
Admix: F1 F2_w : 0.58
Junctions: F2.MALL F3_w : 0.37
Swarm: F2.MALL F4+_w: 0.05
0 2 ABDU 2 1 1 2
Sample: PL613 F1_w : 0.00
Admix: F3.ABDU F2_w : 0.47
Junctions: F3.ABDU F3_w : 0.35
Swarm: F2.ABDU F4+_w: 0.14
6 6 3 2 3 2 1
Sample: PL628 F1_w : 0.00
Admix: F2.ABDU F2_w : 0.28
Junctions: F3 F3_w : 0.64
Swarm: F3 F4+_w: 0.07
3 2 1 3 ABDU/2 2 1
Sample: PL629 F1_w : 0.00
Admix: F1 F2_w : 0.67
Junctions: F2.ABDU F3_w : 0.29
Swarm: F2.ABDU F4+_w: 0.04
2 2 1 1 ABDU ABDU/1 1
Sample: PL639 F1_w : 0.00
Admix: F2.ABDU F2_w : 0.53
Junctions: F2.ABDU F3_w : 0.30
Swarm: F2.ABDU F4+_w: 0.13
7 7 1 3 1 2 1
Sample: PL648 F1_w : 0.00
Admix: F2.MALL F2_w : 0.11
Junctions: F2 F3_w : 0.84
Swarm: F3 F4+_w: 0.06
13 7 8 4 4 4 4
Sample: PL649 F1_w : 0.00
Admix: MALLW−E F2_w : 0.00
Junctions: F4 F3_w : 0.50
Swarm: F5 F4+_w: 0.48
3 2 2 1 ABDU ABDU/2 1
Sample: PL651 F1_w : 0.00
Admix: F2.MALL F2_w : 0.60
Junctions: F2.ABDU F3_w : 0.30
Swarm: F2.ABDU F4+_w: 0.09

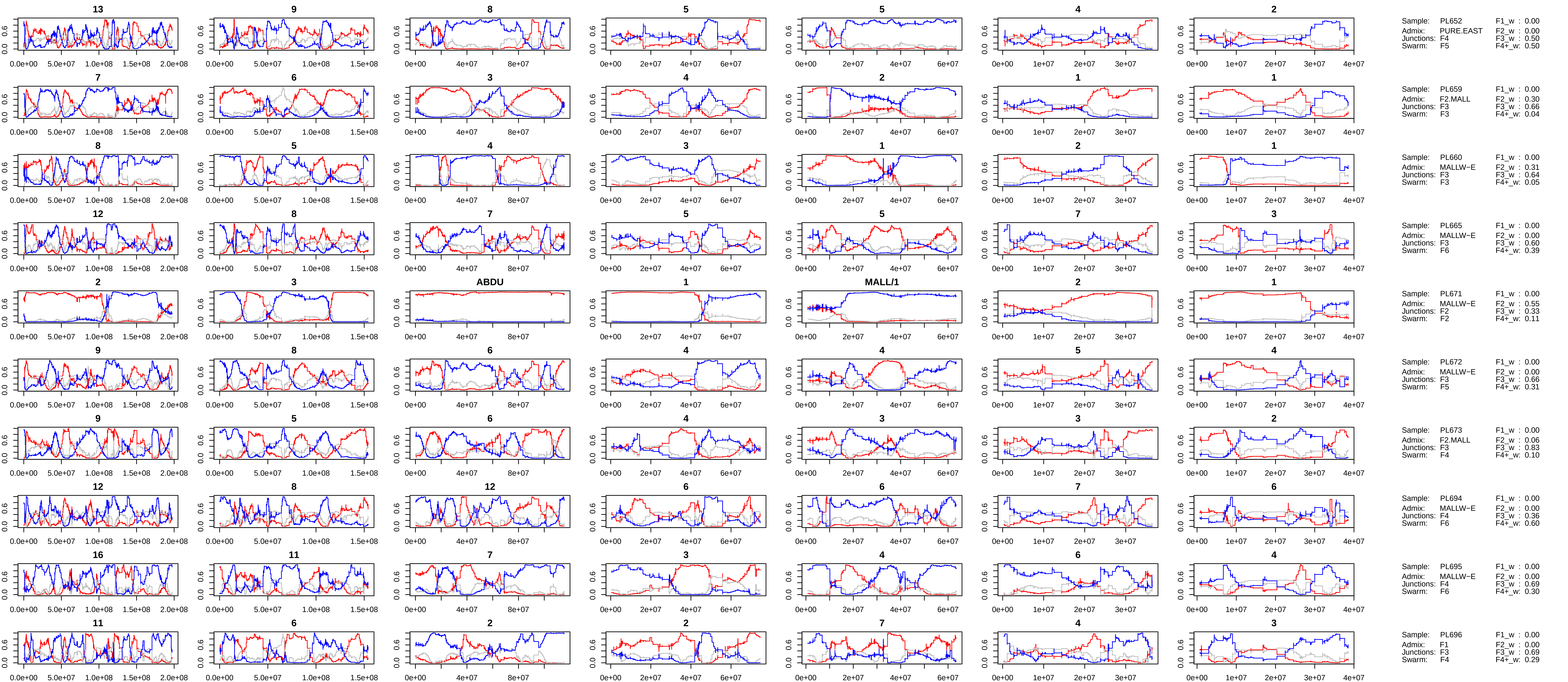
13 9 8 5 5 4 2
Sample: PL652 F1_w : 0.00
Admix: PURE.EAST F2_w : 0.00
Junctions: F4 F3_w : 0.50
Swarm: F5 F4+_w: 0.50
7 6 3 4 2 1 1
Sample: PL659 F1_w : 0.00
Admix: F2.MALL F2_w : 0.30
Junctions: F3 F3_w : 0.66
Swarm: F3 F4+_w: 0.04
8 5 4 3 1 2 1
Sample: PL660 F1_w : 0.00
Admix: MALLW−E F2_w : 0.31
Junctions: F3 F3_w : 0.64
Swarm: F3 F4+_w: 0.05
12 8 7 5 5 7 3
Sample: PL665 F1_w : 0.00
Admix: MALLW−E F2_w : 0.00
Junctions: F3 F3_w : 0.60
Swarm: F6 F4+_w: 0.39
2 3 ABDU 1 MALL/1 2 1
Sample: PL671 F1_w : 0.00
Admix: MALLW−E F2_w : 0.55
Junctions: F2 F3_w : 0.33
Swarm: F2 F4+_w: 0.11
9 8 6 4 4 5 4
Sample: PL672 F1_w : 0.00
Admix: MALLW−E F2_w : 0.00
Junctions: F3 F3_w : 0.66
Swarm: F5 F4+_w: 0.31
9 5 6 4 3 3 2
Sample: PL673 F1_w : 0.00
Admix: F2.MALL F2_w : 0.06
Junctions: F3 F3_w : 0.83
Swarm: F4 F4+_w: 0.10
12 8 12 6 6 7 6
Sample: PL694 F1_w : 0.00
Admix: MALLW−E F2_w : 0.00
Junctions: F4 F3_w : 0.36
Swarm: F6 F4+_w: 0.60
16 11 7 3 4 6 4
Sample: PL695 F1_w : 0.00
Admix: MALLW−E F2_w : 0.00
Junctions: F4 F3_w : 0.69
Swarm: F6 F4+_w: 0.30
11 6 2 2 7 4 3
Sample: PL696 F1_w : 0.00
Admix: F1 F2_w : 0.00
Junctions: F3 F3_w : 0.69
Swarm: F4 F4+_w: 0.29

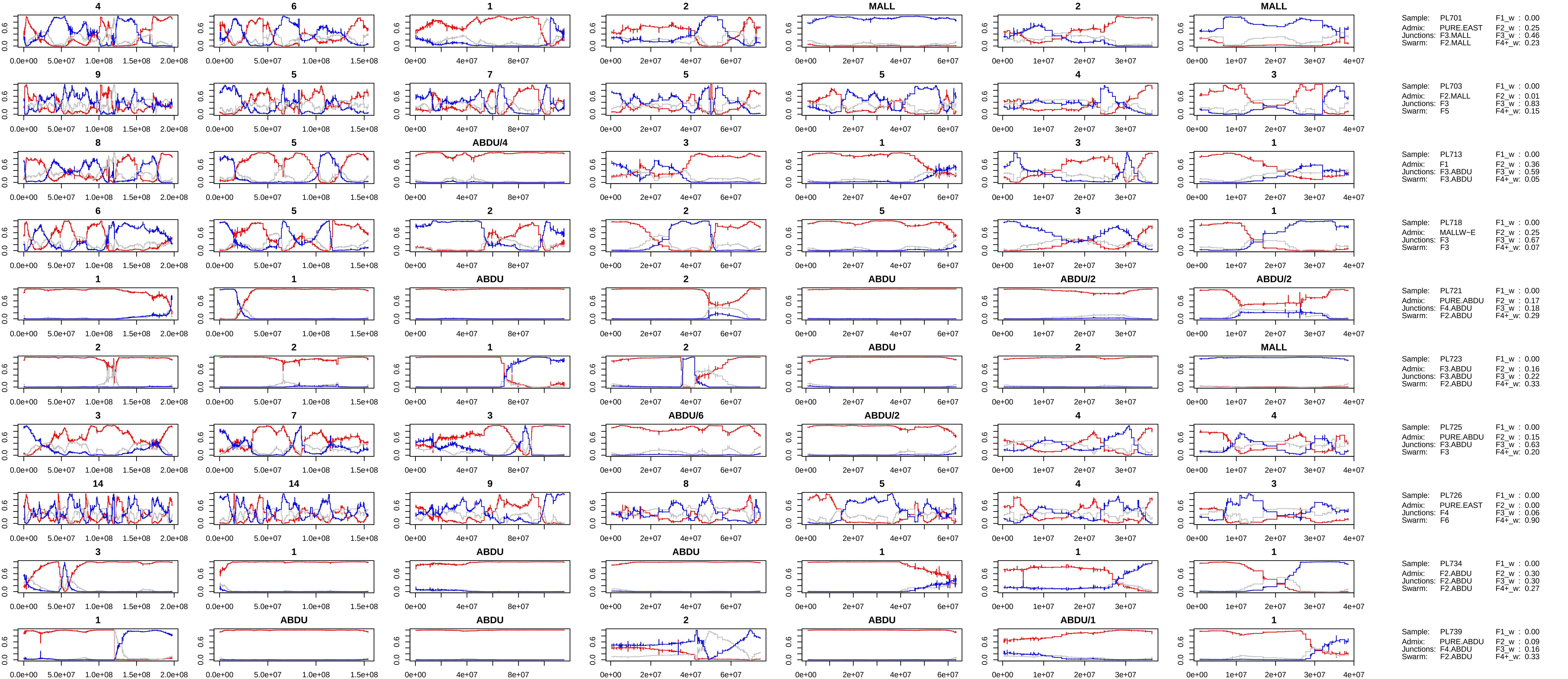

Sample: PL701 F1_w : 0.00
Admix: PURE.EAST F2_w : 0.25
Junctions: F3.MALL F3_w : 0.46
Swarm: F2.MALL F4+_w: 0.23
Sample: PL703 F1_w : 0.00
Admix: F2.MALL F2_w : 0.01
Junctions: F3 F3_w : 0.83
Swarm: F5 F4+_w: 0.15
Sample: PL713 F1_w : 0.00
Admix: F1 F2_w : 0.36
Junctions: F3.ABDU F3_w : 0.59
Swarm: F3.ABDU F4+_w: 0.05
Sample: PL718 F1_w : 0.00
Admix: MALLW−E F2_w : 0.25
Junctions: F3 F3_w : 0.67
Swarm: F3 F4+_w: 0.07
Sample: PL721 F1_w : 0.00
Admix: PURE.ABDU F2_w : 0.17
Junctions: F4.ABDU F3_w : 0.18
Swarm: F2.ABDU F4+_w: 0.29
Sample: PL723 F1_w : 0.00
Admix: F3.ABDU F2_w : 0.16
Junctions: F3.ABDU F3_w : 0.22
Swarm: F2.ABDU F4+_w: 0.33
Sample: PL725 F1_w : 0.00
Admix: PURE.ABDU F2_w : 0.15
Junctions: F3.ABDU F3_w : 0.63
Swarm: F3 F4+_w: 0.20
Sample: PL726 F1_w : 0.00
Admix: PURE.EAST F2_w : 0.00
Junctions: F4 F3_w : 0.06
Swarm: F6 F4+_w: 0.90
Sample: PL734 F1_w : 0.00
Admix: F2.ABDU F2_w : 0.30
Junctions: F2.ABDU F3_w : 0.30
Swarm: F2.ABDU F4+_w: 0.27
Sample: PL739 F1_w : 0.00
Admix: PURE.ABDU F2_w : 0.09
Junctions: F4.ABDU F3_w : 0.16
Swarm: F2.ABDU F4+_w: 0.33

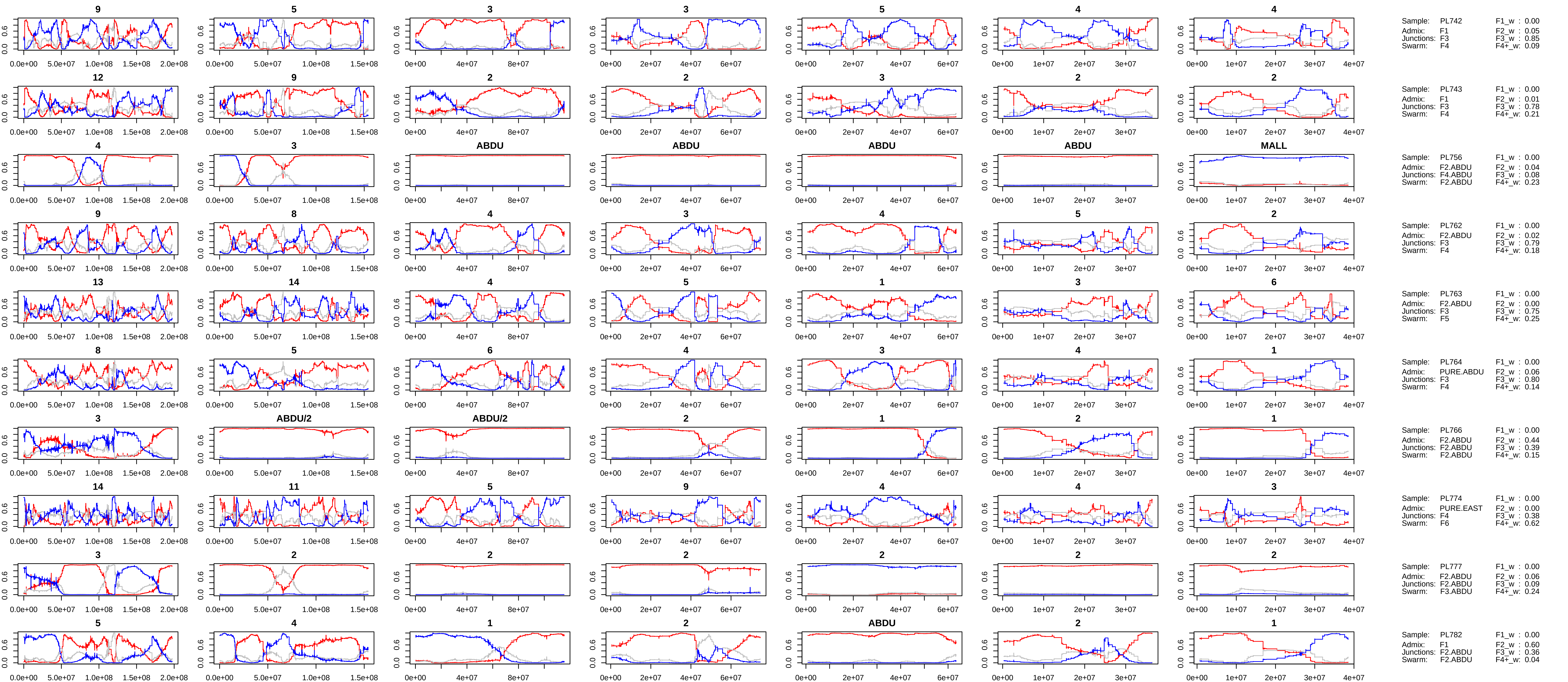
9 5 3 3 5 4 4
Sample: PL742 F1_w : 0.00
Admix: F1 F2_w : 0.05
Junctions: F3 F3_w : 0.85
Swarm: F4 F4+_w: 0.09
12 9 2 2 3 2 2
Sample: PL743 F1_w : 0.00
Admix: F1 F2_w : 0.01
Junctions: F3 F3_w : 0.78
Swarm: F4 F4+_w: 0.21
4 3 ABDU ABDU ABDU ABDU MALL
Sample: PL756 F1_w : 0.00
Admix: F2.ABDU F2_w : 0.04
Junctions: F4.ABDU F3_w : 0.08
Swarm: F2.ABDU F4+_w: 0.23
9 8 4 3 4 5 2
Sample: PL762 F1_w : 0.00
Admix: F2.ABDU F2_w : 0.02
Junctions: F3 F3_w : 0.79
Swarm: F4 F4+_w: 0.18
13 14 4 5 1 3 6
Sample: PL763 F1_w : 0.00
Admix: F2.ABDU F2_w : 0.00
Junctions: F3 F3_w : 0.75
Swarm: F5 F4+_w: 0.25
8 5 6 4 3 4 1
Sample: PL764 F1_w : 0.00
Admix: PURE.ABDU F2_w : 0.06
Junctions: F3 F3_w : 0.80
Swarm: F4 F4+_w: 0.14
3 ABDU/2 ABDU/2 2 1 2 1
Sample: PL766 F1_w : 0.00
Admix: F2.ABDU F2_w : 0.44
Junctions: F2.ABDU F3_w : 0.39
Swarm: F2.ABDU F4+_w: 0.15
14 11 5 9 4 4 3
Sample: PL774 F1_w : 0.00
Admix: PURE.EAST F2_w : 0.00
Junctions: F4 F3_w : 0.38
Swarm: F6 F4+_w: 0.62
3 2 2 2 2 2 2
Sample: PL777 F1_w : 0.00
Admix: F2.ABDU F2_w : 0.06
Junctions: F2.ABDU F3_w : 0.09
Swarm: F3.ABDU F4+_w: 0.24
5 4 1 2 ABDU 2 1
Sample: PL782 F1_w : 0.00
Admix: F1 F2_w : 0.60
Junctions: F2.ABDU F3_w : 0.36
Swarm: F2.ABDU F4+_w: 0.04

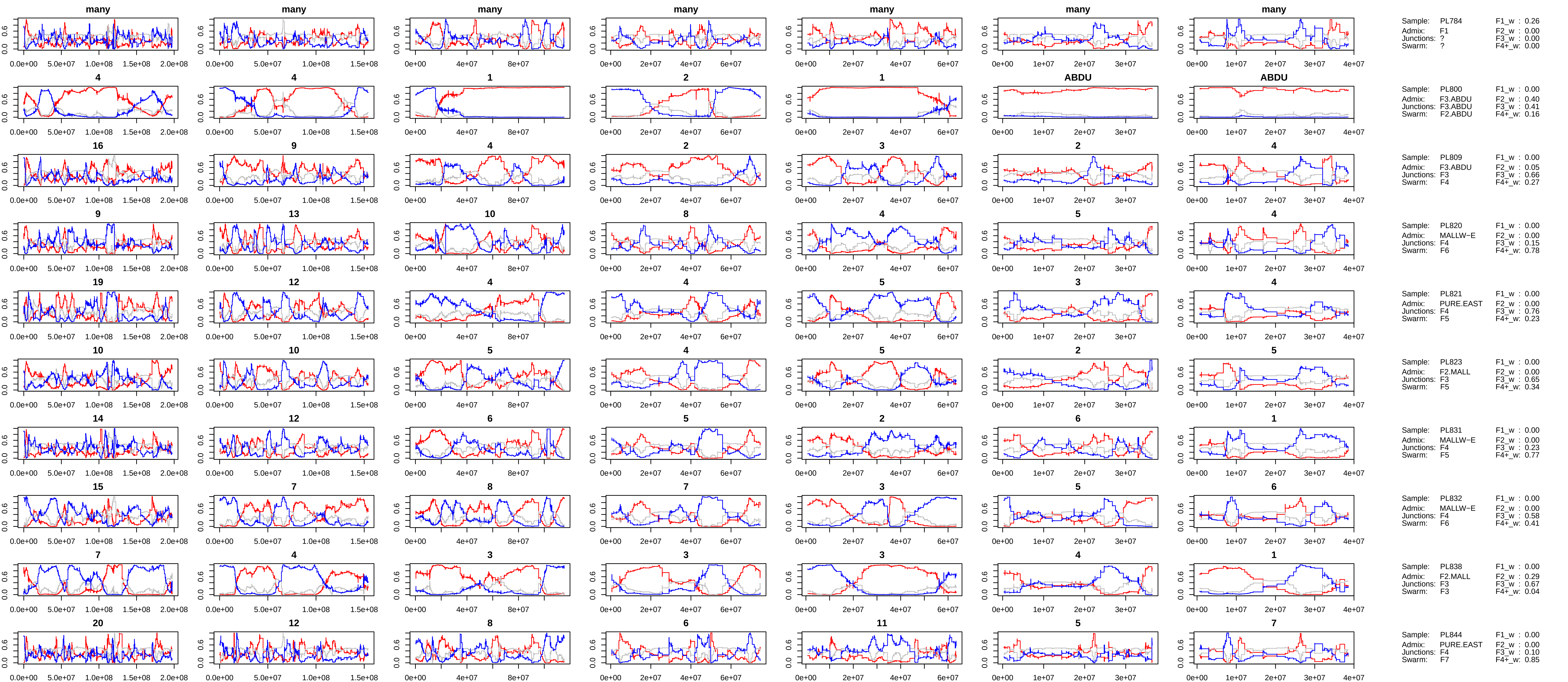

many many many many many many many
Sample: PL784 F1_w : 0.26
Admix: F1 F2_w : 0.00
Junctions: ? F3_w : 0.00
Swarm: ? F4+_w: 0.00
4 4 1 2 1 ABDU ABDU
Sample: PL800 F1_w : 0.00
Admix: F3.ABDU F2_w : 0.40
Junctions: F3.ABDU F3_w : 0.41
Swarm: F2.ABDU F4+_w: 0.16
16 9 4 2 3 2 4
Sample: PL809 F1_w : 0.00
Admix: F3.ABDU F2_w : 0.05
Junctions: F3 F3_w : 0.66
Swarm: F4 F4+_w: 0.27
9 13 10 8 4 5 4
Sample: PL820 F1_w : 0.00
Admix: MALLW−E F2_w : 0.00
Junctions: F4 F3_w : 0.15
Swarm: F6 F4+_w: 0.78
19 12 4 4 5 3 4
Sample: PL821 F1_w : 0.00
Admix: PURE.EAST F2_w : 0.00
Junctions: F4 F3_w : 0.76
Swarm: F5 F4+_w: 0.23
10 10 5 4 5 2 5
Sample: PL823 F1_w : 0.00
Admix: F2.MALL F2_w : 0.00
Junctions: F3 F3_w : 0.65
Swarm: F5 F4+_w: 0.34
14 12 6 5 2 6 1
Sample: PL831 F1_w : 0.00
Admix: MALLW−E F2_w : 0.00
Junctions: F4 F3_w : 0.23
Swarm: F5 F4+_w: 0.77
15 7 8 7 3 5 6
Sample: PL832 F1_w : 0.00
Admix: MALLW−E F2_w : 0.00
Junctions: F4 F3_w : 0.58
Swarm: F6 F4+_w: 0.41
7 4 3 3 3 4 1
Sample: PL838 F1_w : 0.00
Admix: F2.MALL F2_w : 0.29
Junctions: F3 F3_w : 0.67
Swarm: F3 F4+_w: 0.04
20 12 8 6 11 5 7
Sample: PL844 F1_w : 0.00
Admix: PURE.EAST F2_w : 0.00
Junctions: F4 F3_w : 0.10
Swarm: F7 F4+_w: 0.85

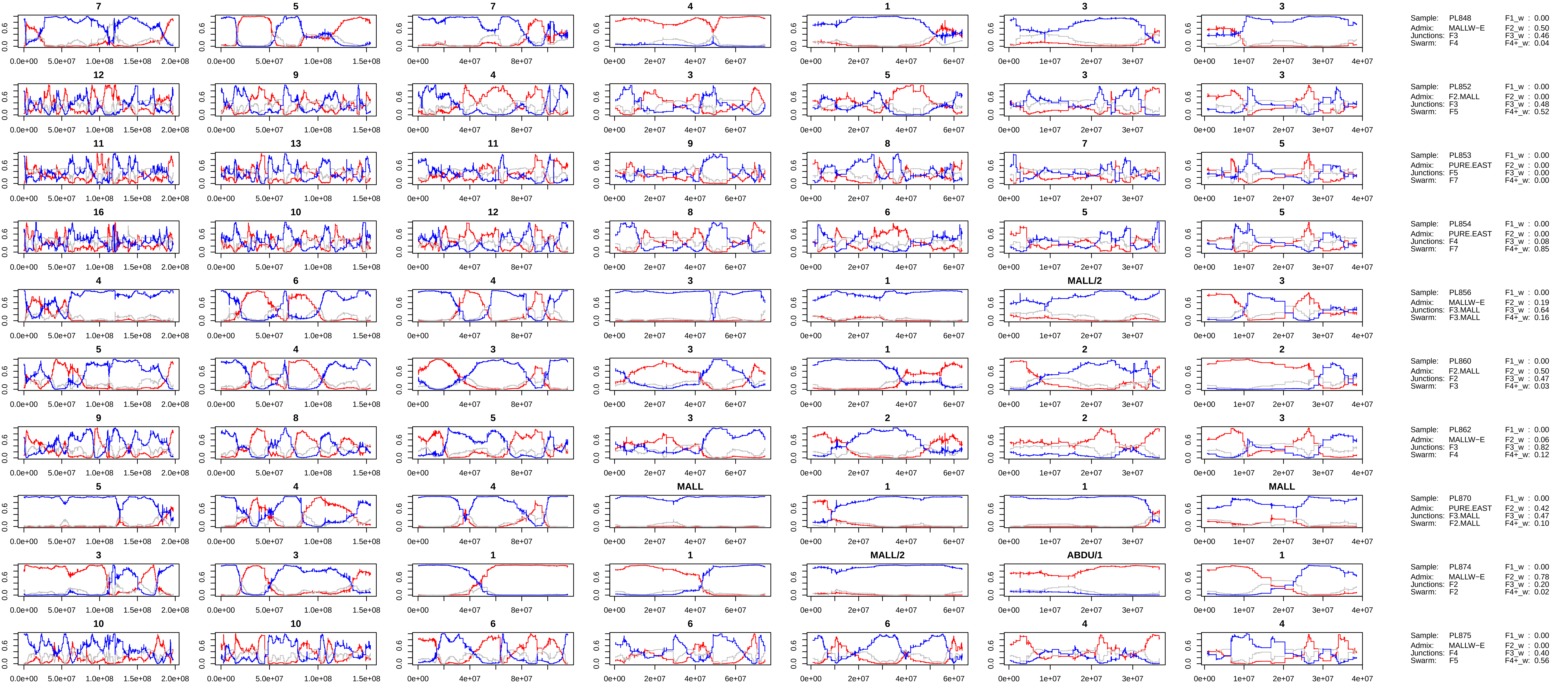
Sample: PL848 F1_w : 0.00
Admix: MALLW−E F2_w : 0.50
Junctions: F3 F3_w : 0.46
Swarm: F4 F4+_w: 0.04
Sample: PL852 F1_w : 0.00
Admix: F2.MALL F2_w : 0.00
Junctions: F3 F3_w : 0.48
Swarm: F5 F4+_w: 0.52
Sample: PL853 F1_w : 0.00
Admix: PURE.EAST F2_w : 0.00
Junctions: F5 F3_w : 0.00
Swarm: F7 F4+_w: 0.00
Sample: PL854 F1_w : 0.00
Admix: PURE.EAST F2_w : 0.00
Junctions: F4 F3_w : 0.08
Swarm: F7 F4+_w: 0.85
Sample: PL856 F1_w : 0.00
Admix: MALLW−E F2_w : 0.19
Junctions: F3.MALL F3_w : 0.64
Swarm: F3.MALL F4+_w: 0.16
Sample: PL860 F1_w : 0.00
Admix: F2.MALL F2_w : 0.50
Junctions: F2 F3_w : 0.47
Swarm: F3 F4+_w: 0.03
Sample: PL862 F1_w : 0.00
Admix: MALLW−E F2_w : 0.06
Junctions: F3 F3_w : 0.82
Swarm: F4 F4+_w: 0.12
Sample: PL870 F1_w : 0.00
Admix: PURE.EAST F2_w : 0.42
Junctions: F3.MALL F3_w : 0.47
Swarm: F2.MALL F4+_w: 0.10
Sample: PL874 F1_w : 0.00
Admix: MALLW−E F2_w : 0.78
Junctions: F2 F3_w : 0.20
Swarm: F2 F4+_w: 0.02
Sample: PL875 F1_w : 0.00
Admix: MALLW−E F2_w : 0.00
Junctions: F4 F3_w : 0.40
Swarm: F5 F4+_w: 0.56

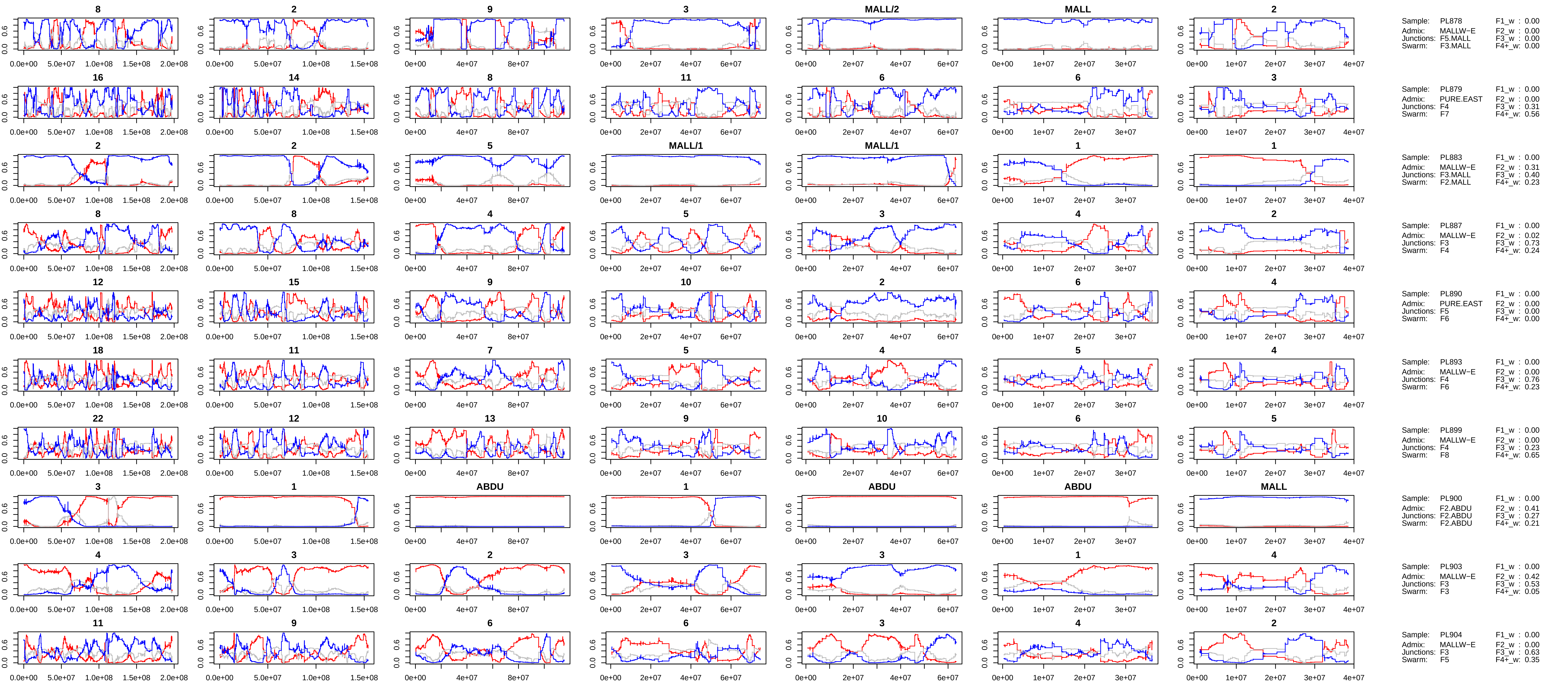
8
2
9
3
MALL/2
MALL
2
Sample: PL878 F1_w : 0.00
Admix: MALLW−E F2_w : 0.00
Junctions: F5.MALL F3_w : 0.00
Swarm: F3.MALL F4+_w: 0.00
16
14
8
11
6
6
3
Sample: PL879 F1_w : 0.00
Admix: PURE.EAST F2_w : 0.00
Junctions: F4 F3_w : 0.31
Swarm: F7 F4+_w: 0.56
2
2
5
MALL/1
MALL/1
1
1
Sample: PL883 F1_w : 0.00
Admix: MALLW−E F2_w : 0.31
Junctions: F3.MALL F3_w : 0.40
Swarm: F2.MALL F4+_w: 0.23
8
8
4
5
3
4
2
Sample: PL887 F1_w : 0.00
Admix: MALLW−E F2_w : 0.02
Junctions: F3 F3_w : 0.73
Swarm: F4 F4+_w: 0.24
12
15
9
10
2
6
4
Sample: PL890 F1_w : 0.00
Admix: PURE.EAST F2_w : 0.00
Junctions: F5 F3_w : 0.00
Swarm: F6 F4+_w: 0.00
18
11
7
5
4
5
4
Sample: PL893 F1_w : 0.00
Admix: MALLW−E F2_w : 0.00
Junctions: F4 F3_w : 0.76
Swarm: F6 F4+_w: 0.23
22
12
13
9
10
6
5
Sample: PL899 F1_w : 0.00
Admix: MALLW−E F2_w : 0.00
Junctions: F4 F3_w : 0.23
Swarm: F8 F4+_w: 0.65
3
1
ABDU
1
ABDU
ABDU
MALL
Sample: PL900 F1_w : 0.00
Admix: F2.ABDU F2_w : 0.41
Junctions: F2.ABDU F3_w : 0.27
Swarm: F2.ABDU F4+_w: 0.21
4
3
2
3
3
1
4
Sample: PL903 F1_w : 0.00
Admix: MALLW−E F2_w : 0.42
Junctions: F3 F3_w : 0.53
Swarm: F3 F4+_w: 0.05
11
9
6
6
3
4
2
Sample: PL904 F1_w : 0.00
Admix: MALLW−E F2_w : 0.00
Junctions: F3 F3_w : 0.63
Swarm: F5 F4+_w: 0.35

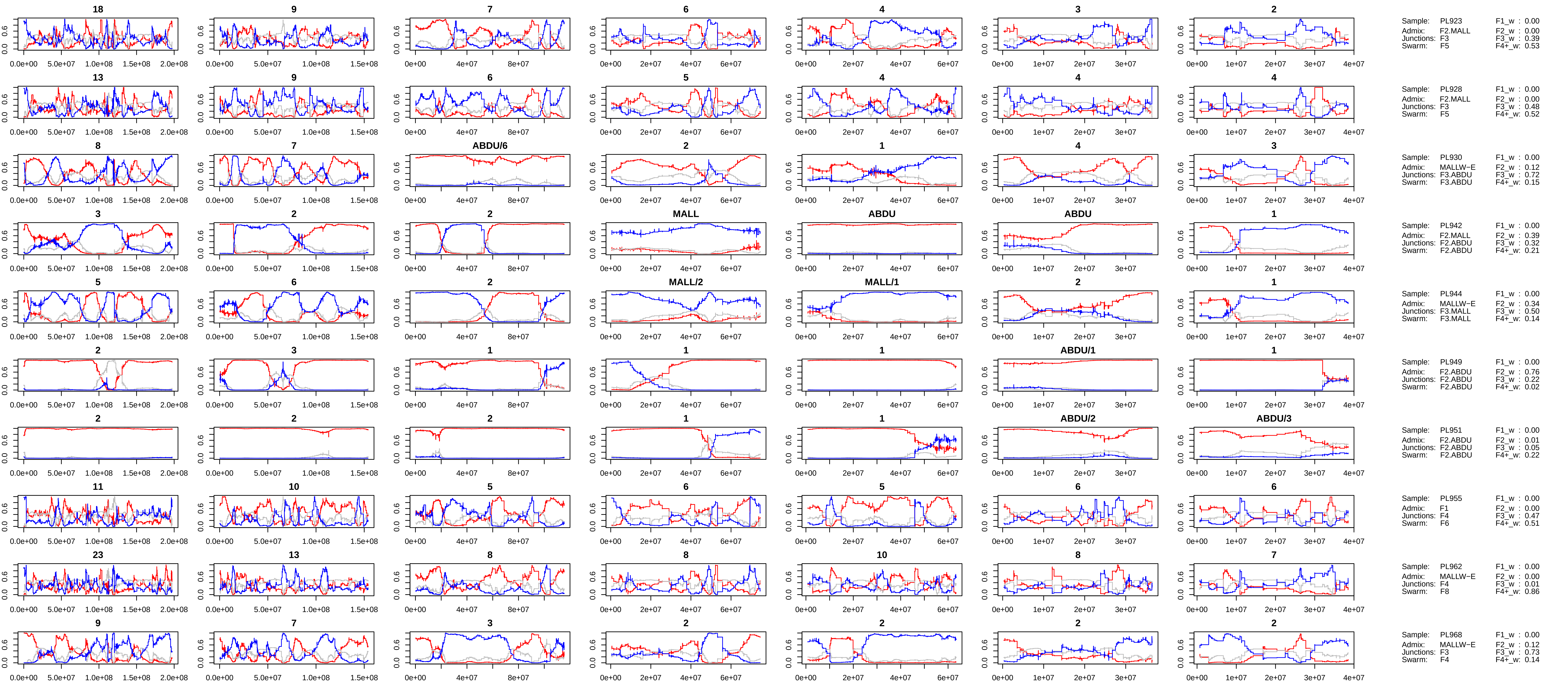
18 9 7 6 4 3 2
Sample: PL923 F1_w : 0.00
Admix: F2.MALL F2_w : 0.00
Junctions: F3 F3_w : 0.39
Swarm: F5 F4+_w: 0.53
13 9 6 5 4 4 4
Sample: PL928 F1_w : 0.00
Admix: F2.MALL F2_w : 0.00
Junctions: F3 F3_w : 0.48
Swarm: F5 F4+_w: 0.52
8 7 ABDU/6 2 1 4 3
Sample: PL930 F1_w : 0.00
Admix: MALLW−E F2_w : 0.12
Junctions: F3.ABDU F3_w : 0.72
Swarm: F3.ABDU F4+_w: 0.15
3 2 2 MALL ABDU ABDU 1
Sample: PL942 F1_w : 0.00
Admix: F2.MALL F2_w : 0.39
Junctions: F2.ABDU F3_w : 0.32
Swarm: F2.ABDU F4+_w: 0.21
5 6 2 MALL/2 MALL/1 2 1
Sample: PL944 F1_w : 0.00
Admix: MALLW−E F2_w : 0.34
Junctions: F3.MALL F3_w : 0.50
Swarm: F3.MALL F4+_w: 0.14
2 3 1 1 1 ABDU/1 1
Sample: PL949 F1_w : 0.00
Admix: F2.ABDU F2_w : 0.76
Junctions: F2.ABDU F3_w : 0.22
Swarm: F2.ABDU F4+_w: 0.02
2 2 2 1 1 ABDU/2 ABDU/3
Sample: PL951 F1_w : 0.00
Admix: F2.ABDU F2_w : 0.01
Junctions: F2.ABDU F3_w : 0.05
Swarm: F2.ABDU F4+_w: 0.22
11 10 5 6 5 6 6
Sample: PL955 F1_w : 0.00
Admix: F1 F2_w : 0.00
Junctions: F4 F3_w : 0.47
Swarm: F6 F4+_w: 0.51
23 13 8 8 10 8 7
Sample: PL962 F1_w : 0.00
Admix: MALLW−E F2_w : 0.00
Junctions: F4 F3_w : 0.01
Swarm: F8 F4+_w: 0.86
9 7 3 2 2 2 2
Sample: PL968 F1_w : 0.00
Admix: MALLW−E F2_w : 0.12
Junctions: F3 F3_w : 0.73
Swarm: F4 F4+_w: 0.14

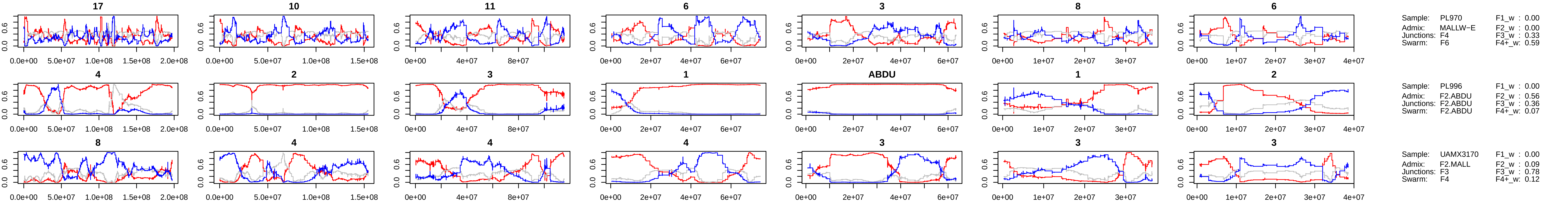
17
10
11
6
3
8
6
Sample: PL970
Admix: MALLW−E
Junctions: F4
Swarm: F6
F1_w : 0.00
F2_w : 0.00
F3_w : 0.33
F4+_w: 0.59
4
2
3
1
ABDU
1
2
Sample: PL996
Admix: F2.ABDU
Junctions: F2.ABDU
Swarm: F2.ABDU
F1_w : 0.00
F2_w : 0.56
F3_w : 0.36
F4+_w: 0.07
8
4
4
4
3
3
3
Sample: UAMX3170
Admix: F2.MALL
Junctions: F3
Swarm: F4
F1_w : 0.00
F2_w : 0.09
F3_w : 0.78
F4+_w: 0.12
