# Supplementary material for: Identifying hybrids & the genomics of hybridization: Mallards & American black ducks of Eastern North America
Source: Ecol Evol. 2019 Feb 27;9(6):3470–90. doi: 10.1002/ece3.4981 (PMC6434578; doi:10.1002/ece3.4981)
Supplement: Supplementary file 12 [file ECE3-9-3470-s013.docx]

**Supplementary Material Table S4**. Nucleotide diversity (π) and Watterson’s θ estimated and scaled to total base-pairs by chromosome using the R program PopGenome ([Pfeifer et al. 2014](#_ENREF_68)) across chromosomally concatenated ddRAD marker datasets for genetically vetted (also see Fig. 2) American black ducks (ABDU), western mallards (MALL.W), and non-western mallards (MALL.NonW).

|  |  | **Nucleotide diversity (π)** | | | **Watterson’s θ** | | |
| --- | --- | --- | --- | --- | --- | --- | --- |
|  | Nucleotides | ABDU | MALL.W | MALL.NonW | ABDU | MALL.W | MALL.NonW |
| Z-Chromosome | 19,873 | 0.0024 | 0.0027 | 0.0026 | 0.0047 | 0.0047 | 0.0029 |
| mtDNA | 625 | 0.0062 | 0.014 | 0.0090 | 0.011 | 0.012 | 0.011 |
| Chromosome 1 | 51,280 | 0.0055 | 0.0054 | 0.0050 | 0.010 | 0.0089 | 0.0063 |
| Chromosome 2 | 8,283 | 0.0058 | 0.0056 | 0.0049 | 0.012 | 0.011 | 0.0067 |
| Chromosome 3 | 7,291 | 0.0059 | 0.0059 | 0.0052 | 0.012 | 0.011 | 0.0072 |
| Chromosome 4 | 9,005 | 0.0058 | 0.0055 | 0.0050 | 0.010 | 0.0095 | 0.0067 |
| Chromosome 5 | 9,476 | 0.0066 | 0.0066 | 0.0056 | 0.014 | 0.014 | 0.0089 |
| Chromosome 6 | 8,936 | 0.0070 | 0.0067 | 0.0063 | 0.015 | 0.014 | 0.0095 |
| Chromosome 7 | 8,911 | 0.0064 | 0.0063 | 0.0052 | 0.012 | 0.011 | 0.0070 |
| Chromosome 8 | 7,160 | 0.0072 | 0.0070 | 0.0070 | 0.014 | 0.013 | 0.010 |
| Chromosome 9 | 7,152 | 0.0085 | 0.0087 | 0.0076 | 0.016 | 0.016 | 0.011 |
| Chromosome 10 | 7,007 | 0.0062 | 0.0060 | 0.0053 | 0.014 | 0.014 | 0.010 |
| Chromosome 11 | 40,480 | 0.0072 | 0.0071 | 0.0069 | 0.010 | 0.010 | 0.0057 |
| Chromosome 12 | 4,923 | 0.0060 | 0.0057 | 0.0058 | 0.013 | 0.011 | 0.0082 |
| Chromosome 13 | 6,587 | 0.0087 | 0.0086 | 0.0081 | 0.012 | 0.011 | 0.0081 |
| Chromosome 14 | 4,033 | 0.0094 | 0.0092 | 0.0084 | 0.014 | 0.012 | 0.0072 |
| Chromosome 15 | 2,460 | 0.0063 | 0.0060 | 0.0058 | 0.015 | 0.015 | 0.010 |
| Chromosome 16 | 3,310 | 0.0093 | 0.0094 | 0.0089 | 0.017 | 0.017 | 0.011 |
| Chromosome 18 | 5,049 | 0.011 | 0.011 | 0.010 | 0.016 | 0.015 | 0.011 |
| Chromosome 19 | 291 | 0.0088 | 0.0090 | 0.0077 | 0.010 | 0.012 | 0.010 |
| Chromosome 20 | 4,201 | 0.0079 | 0.0077 | 0.0066 | 0.015 | 0.014 | 0.0094 |
| Chromosome 21 | 1,996 | 0.0074 | 0.0073 | 0.0068 | 0.015 | 0.014 | 0.0084 |
| Chromosome 22 | 2,746 | 0.0080 | 0.0079 | 0.0061 | 0.013 | 0.012 | 0.0075 |
| Chromosome 23 | 34,471 | 0.011 | 0.011 | 0.0094 | 0.011 | 0.010 | 0.0062 |
| Chromosome 24 | 20,597 | 0.011 | 0.011 | 0.0099 | 0.010 | 0.0091 | 0.0058 |
| Chromosome 25 | 25,308 | 0.011 | 0.011 | 0.010 | 0.011 | 0.010 | 0.0065 |
| Chromosome 26 | 15,978 | 0.0093 | 0.011 | 0.010 | 0.010 | 0.010 | 0.0069 |
| Chromosome 27 | 11,330 | 0.0092 | 0.0091 | 0.0080 | 0.011 | 0.010 | 0.0075 |
| Chromosome 28 | 12,201 | 0.0080 | 0.0085 | 0.0068 | 0.011 | 0.010 | 0.0075 |
| Chromosome 29 | 10,545 | 0.0081 | 0.0083 | 0.0075 | 0.014 | 0.013 | 0.0084 |
| Unknown Markers | 31,637 | 0.0082 | 0.0083 | 0.0075 | 0.013 | 0.012 | 0.0082 |
