# Supplementary material for: Identifying hybrids & the genomics of hybridization: Mallards & American black ducks of Eastern North America
Source: Ecol Evol. 2019 Feb 27;9(6):3470–90. doi: 10.1002/ece3.4981 (PMC6434578; doi:10.1002/ece3.4981)
Supplement: Supplementary file 13 [file ECE3-9-3470-s014.docx]

**Supplementary Materials Table S3**. Hybrid status assignment based on different reference panels and agreement with ADMIXTURE analyses for: (A) Default Panel under a unidirectional backcrossing scheme, (B) Default Panel under a hybrid swarm scenario, (C) Panel based on samples with at most one recombination, and (D) a panel comprising individuals that are 100% pure based on recombination analyses.

**(A) Default panel**

|  |  | F1 | F2 | F3 | F4+ | Match (%) |
| --- | --- | --- | --- | --- | --- | --- |
| Admixture | F1 | **0** | 2 | 15 | 10 | 0 |
|  | F2 | 0 | **15** | 14 | 10 | 38 |
|  | F3 | 0 | 0 | **4** | 2 | 67 |
|  | F4+ | 0 | 8 | 29 | **34** | 48 |
|  | Total | 0 | 25 | 62 | 56 | 37 |

**(B) Default panel, hybrid swarm**

|  |  | F1 | F2 | F3 | F4+ | Match (%) |
| --- | --- | --- | --- | --- | --- | --- |
| Admixture | F1 | **0** | 10 | 5 | 12 | 0 |
|  | F2 | 0 | **17** | 10 | 12 | 44 |
|  | F3 | 0 | 4 | **0** | 2 | 0 |
|  | F4+ | 0 | 20 | 14 | **37** | 52 |
|  | Overall | 0 | 51 | 29 | 63 | 38 |

**(C) Panel including individuals with at most one recombination**

|  |  | F1 | F2 | F3 | F4+ | Match (%) |
| --- | --- | --- | --- | --- | --- | --- |
| Admixture | F1 | **0** | 2 | 3 | 22 | 0 |
|  | F2 | 0 | **3** | 10 | 26 | 8 |
|  | F3 | 0 | 0 | **0** | 6 | 0 |
|  | F4+ | 0 | 9 | 24 | **38** | 54 |
|  | Overall | 0 | 14 | 37 | 92 | 29 |

**(D) Panel only including 100% genetically pure individuals**

|  |  | F1 | F2 | F3 | F4+ | Match (%) |
| --- | --- | --- | --- | --- | --- | --- |
| Admixture | F1 | **0** | 0 | 0 | 27 | 0 |
|  | F2 | 0 | **1** | 3 | 35 | 3 |
|  | F3 | 0 | 0 | **0** | 6 | 0 |
|  | F4+ | 0 | 3 | 9 | **59** | 83 |
|  | Overall | 0 | 4 | 12 | 127 | 42 |
