# Supplementary material for: Identifying hybrids & the genomics of hybridization: Mallards & American black ducks of Eastern North America
Source: Ecol Evol. 2019 Feb 27;9(6):3470–90. doi: 10.1002/ece3.4981 (PMC6434578; doi:10.1002/ece3.4981)
Supplement: Supplementary file 14 [file ECE3-9-3470-s002.docx]

**I. SIMULATING EXPECTATIONS UNDER A SINGLE PARENTAL BACKCROSSING SCENARIO**

Following Janzen et al (2018), new junctions are formed when a crossover takes place at a site that is heterogenic for ancestry. Under a scenario where backcrossing occurs into a single parental taxa, the first generation offspring are full hybrids of both parents, inheriting one chromosome from one parental species, and one chromosome from the other. Therefore, we define the number of junctions of the F_1_ as 0, and the heterozygosity of the F_1_ as 1 (on all sites along the genome individuals are heterozygous for ancestry). This differs from Janzen et al. (2018) whom assumed a randomly mating hybrid swarm, where initial frequencies followed Hardy-Weinberg proportions and the initial heterozygosity for a first generation offspring of two randomly mating ancestors would be 0.5. Subsequently, upon backcrossing, a new individual inherits one chromosome from the ancestor, which by definition does not have any junctions, and one chromosome from an individual from the previous generation. The number of junctions in the recombined chromosome is then the expected number of junctions in the previous generation, plus the heterozygosity times the size of the chromosome in Morgan. Thus, the expected number of junctions at time t+1 is then:

$$E\left[ J_{t+1} \right]= \frac{0+ \left( J_{t}+{CH}_{t} \right)}{2} (1)$$

Where *J_t_* is the number of junctions at generation t and *C* is the size in Morgan of the chromosome, which corresponds to a Poisson distributed number of crossovers per meiosis with rate C. *H_t_* is the heterozygosity at t, and we divide by 2 to average over the two chromosomes involved. Because there is continuous backcrossing, *H_t_* has a simple relationship such that each generation the average heterozygosity is halved and we find *H_t_ = 2^-t^* . Then, the solution of (1) is given by:

$$E\left[ J_{t} \right]=C (t-1) 2^{-(t-1)} (2)$$

Where *t* represents the generation, e.g *F_1_* is *t = 1*. Comparing simulation results with our expectation, we find a very close fit between the two (Imbedded Fig. 1).


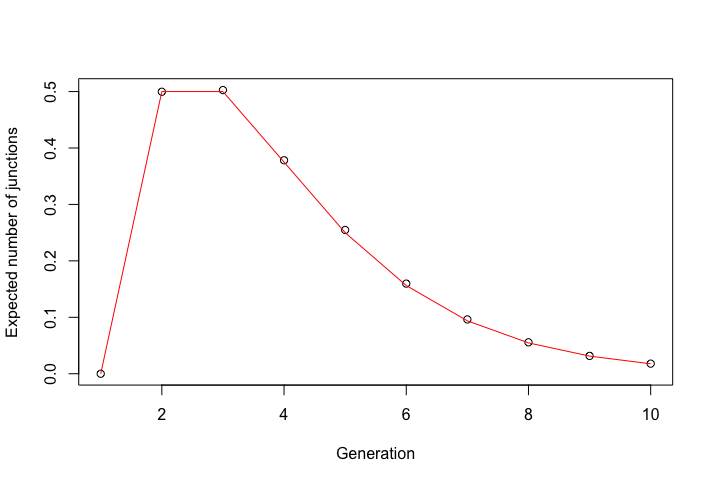


**Figure 1**. Expected number of junctions over time for a population of 1000 individuals, in a chromosome of 1 Morgan (C = 1). 1000 markers were used (R = 1000). Points indicate the average number of junctions across 100 replicate simulations, the red line indicates the expectation following equation 2.

Next, to obtain the fraction of genomic material identical to the backcrossing parent (main text Fig. 3A), we only need to calculate the total amount of homozygosity across the genome; because due to the backcrossing scheme, one chromosome is already fully identical to the backcrossing parent, we are only interested in those areas in the other chromosome that are not identical to the backcrossing parent, which results in local heterozygosity. The total amount of genomic content then identical to the backcrossing parent is then equivalent to the total homozygosity, or alternatively, to one minus the heterozygosity:

*H_o_ = 1 - 2^-t^*  (3)

Here, in contrast to Janzen et al 2018, we have ignored effects of population size and number of markers. Whereas in Janzen et al. 2018 the crossing scheme generates potential fixation of sites, the backcrossing scheme implored here does not impact the expected heterozygosity: over time the finite population size does not contribute to increased fixation of loci. A finite number of markers might potentially impact results, but because typically R >> Jt, any limiting effects on having a finite number of markers can also be safely ignored. Thus, since we know that per definition, one chromosome contains no junctions (the chromosome inherited from the parent during the backcross), the expected number of junctions on the hybrid chromosome is given by:

$$E\left[ J_{t} \right]=2C (t-1) 2^{-(t-1)} (4)$$

***Interesting Properties***: Because the average heterozygosity is always decreasing, and the number of junctions after F3 is also always decreasing, we observe that individuals are becoming increasingly homozygous, and start to resemble the parents. Although F10 have almost the same number of junctions as the F1, the F1 are completely heterozygous, whilst in contrast, the F10 are almost completely homozygous (Embedded Table 1).

More interesting perhaps is the full distribution of junctions within the population. This provides us with an expectation of the number of discrete junctions, given the number of generations, and the size of the chromosome in Morgan. As the number of generations increases, we loose tractability of this problem, as the number of combinations in which to obtain the same number of junctions within the given number of generations increases exponentially. Therefore, we use simulations to obtain expected frequencies. Because we know that population size and number of markers are of little effect, we use a modest population size (N = 1,000) and a modest number of markers (R = 1,000) to simulate the accumulation of junctions. Table S1 shows the results for C = 1, but a full table for sizes 3.17, 2.26, 1.12, 0.93, 0.79, 1.20, and 0.98 Morgan (Huang *et al.* 2006) for chromosomes 1-7 respectively can be found in the Supplementary Material.

**Table 1**. Rows indicate separate generations, columns indicate the number of junctions. Numbers in the cells represent the frequency of individuals with that number of junctions, at that specific generation.

| Generation | 0 | 1 | 2 | 3 | 4 | 5 | 6 | 7 |
| --- | --- | --- | --- | --- | --- | --- | --- | --- |
| 1 | 1.000 | 0 | 0 | 0 | 0 | 0 | 0 | 0 |
| 2 | 0.370 | 0.367 | 0.183 | 0.061 | 0.015 | 0.003 | 0 | 0 |
| 3 | 0.441 | 0.266 | 0.193 | 0.067 | 0.025 | 0.006 | 0.002 | 0 |
| 4 | 0.607 | 0.156 | 0.163 | 0.043 | 0.024 | 0.005 | 0.002 | 0 |
| 5 | 0.746 | 0.086 | 0.122 | 0.023 | 0.018 | 0.003 | 0.002 | 0 |
| 6 | 0.844 | 0.046 | 0.085 | 0.011 | 0.012 | 0.002 | 0.001 | 0 |
| 7 | 0.907 | 0.024 | 0.055 | 0.005 | 0.007 | 0.001 | 0 | 0 |
| 8 | 0.946 | 0.013 | 0.034 | 0.003 | 0.004 | 0.001 | 0 | 0 |
| 9 | 0.969 | 0.007 | 0.020 | 0.002 | 0.001 | 0 | 0 | 0 |
| 10 | 0.983 | 0.005 | 0.011 | 0.001 | 0 | 0 | 0 | 0 |

If we study Table S1, we observe that from F7 onwards, more than 90% of the population has on average 0 junctions, across both chromosomes. Furthermore, if there are 2 or more junctions observed, it becomes hard to estimate the age of the individual, as multiple generations have very similar frequencies of chromosomes with 2 or more junctions, making it hard to pick a specific generation that dominates. This could potentially explain the bias in inference of the data towards F3 individuals, as we see that for an individual with 2-7 junctions, the highest frequency is always observed in the F3 generation

**II. ASSIGNING HYBRID GENERATION ACROSS EMPIRICAL DATA**

To calculate the likelihood of an individual being of generation *t*, given a number of junctions, we require an expectation of the distribution of junctions, given *t*. Unfortunately, the expectation of the variance in the number of junctions is a particularly difficult problem to solve; although for a random mating scheme some approximations exist ([e.g., Chapman and Thompson 2002](#_ENREF_13)), such expressions are lacking for a backcrossing scheme. In general, the probability of an individual being of generation *t* is given by:

$$P\left( t \right)= \prod_{i=1}^{7} P\left( J_{i},t, C_{i} \right)$$

Where *J_i_* is the number of junctions at chromosome *i*, $P\left( J_{i},t, C_{i} \right)$ is the probability of observing *J_i_* junctions at time *t* given a chromosome of size $C_{i}$in Morgan. In the absence of an expression for $P\left( J_{i},t, C_{i} \right)$ we can still obtain a good approximation by using the frequency of observing *J_i_* junctions at time *t* given a chromosome of size $C_{i}$in Morgan in simulations. Hence, we obtained observed frequencies $f\left( J_{i,}t,C_{i} \right)$ by performing simulations, where we scored the observed number of junctions per generation, for the seven different chromosome sizes, across 1,000 replicates, with a population size of 10,000 individuals. Variation in frequency across replicates was low (main text Supplementary Materials Table S2), indicating that our approximation is very accurate. Then, given observed frequencies, the log likelihood of an individual being of generation *t*, is given by:

$$logP\left( t \right)= \sum_{i=1}^{7} \log f(J_{i,}t,C_{i})$$
